# IL-6 deficiency accelerates cerebral cryptococcosis and alters glial cell responses

**DOI:** 10.1186/s12974-024-03237-x

**Published:** 2024-09-27

**Authors:** Marta Reguera-Gomez, Melissa E. Munzen, Mohamed F. Hamed, Claudia L. Charles-Niño, Luis R. Martinez

**Affiliations:** 1 Department of Oral Biology, University of Florida College of Dentistry, 1395 Center Drive, DG-48, P.O. Box 100424, Gainesville, FL 32610 USA; 2 Department of Pathology, Faculty of Veterinary Medicine, Mansoura University, Mansoura, Egypt; 3 Emerging Pathogens Institute, Gainesville, FL USA; 4 Center for Immunology and Transplantation, Gainesville, FL USA; 5 Center for Translational Research in Neurodegenerative Disease, University of Florida, Gainesville, FL USA

**Keywords:** Astrocytes, *Cryptococcus neoformans*, CNS, IL-6, Immunity, Microglia, Phagocytosis

## Abstract

*Cryptococcus neoformans* (*Cn*) is an opportunistic encapsulated fungal pathogen that causes life-threatening meningoencephalitis in immunosuppressed individuals. Since IL-6 is important for blood-brain barrier support and its deficiency has been shown to facilitate *Cn* brain invasion, we investigated the impact of IL-6 on systemic *Cn* infection in vivo, focusing on central nervous system (CNS) colonization and glial responses, specifically microglia and astrocytes. IL-6 knock-out (IL-6^−/−^) mice showed faster mortality than C57BL/6 (Wild-type) and IL-6^−/−^ supplemented with recombinant IL-6 (rIL-6; 40 pg/g/day) mice. Despite showing early lung inflammation but no major histological differences in pulmonary cryptococcosis progression among the experimental groups, IL-6^−/−^ mice had significantly higher blood and brain tissue fungal burden at 7-days post infection. Exposure of cryptococci to rIL-6 in vitro increased capsule growth. In addition, IL-6^−/−^ brains were characterized by an increased dystrophic microglia number during *Cn* infection, which are associated with neurodegeneration and senescence. In contrast, the brains of IL-6-producing or -supplemented mice displayed high numbers of activated and phagocytic microglia, which are related to a stronger anti-cryptococcal response or tissue repair. Likewise, culture of rIL-6 with microglia-like cells promoted high fungal phagocytosis and killing, whereas IL-6 silencing in microglia decreased fungal phagocytosis. Lastly, astrogliosis was high and moderate in infected brains removed from Wild-type and IL-6^−/−^ supplemented with rIL-6 animals, respectively, while minimal astrogliosis was observed in IL-6^−/−^ tissue, highlighting the potential of astrocytes in containing and combating cryptococcal infection. Our findings suggest a critical role for IL-6 in *Cn* CNS dissemination, neurocryptococcosis development, and host defense.

**Supplementary Information:**

The online version contains supplementary material available at 10.1186/s12974-024-03237-x.

## Introduction

*Cryptococcus neoformans* (*Cn*) is an encapsulated yeast-like fungus that causes opportunistic infection mostly in immunocompromised patients and occasionally in healthy individuals. Most cases are reported in sub-Saharan Africa where HIV is highly prevalent and access to optimal anti-cryptococcal therapy is inadequate. *Cn* establishes infection in the lungs upon inhalation of fungal spores or desiccated yeasts [1], enters the bloodstream during immunosuppression, and invades the central nervous system (CNS) transcellularly [2], paracellularly [3], or using phagocytes as a Trojan horse [4] typically resulting in life-threatening meningoencephalitis. Cerebral cryptococcosis is the third most common CNS infection in patients with HIV/AIDS [5]. The mortality rate of *Cn* infection is high (~ 74%), accounting for 112,000 deaths annually worldwide [6]. The polysaccharide capsule is a major contributor to *Cn* virulence [7]. Glucuronoxylomannan (GXM), the main capsular component, is extensively secreted and accumulates considerably in serum and cerebrospinal fluid (CSF) during infection [7, 8], contributing significantly to *Cn* pathogenesis [9]. For instance, GXM stimulates HIV proliferation [10] and weakens the host immunity by interfering with phagocytosis, antigen presentation, leukocyte migration, and specific antibody (Ab) responses [9].

Anti-cryptococcal response in the CNS is weak or delayed compared to that in peripheral organs, suggesting that effective immunity may involve the activation of T cells and their eventual entry into the CNS [11]. Astrocytic proliferation and microglial activation in association with neuronal damage and reduced cellular repair are characteristics of cryptococcal meningoencephalitis [12]. Post-mortem neuropathological examinations of patients’ brains have demonstrated that *Cn* GXM is ingested and localizes inside of microglia [13, 14]. Intracellular and extracellular defense against fungi by microglia depends on cytokine release, such as interferon (IFN)-γ, complement activation [15], and opsonization of antigens [16]. For instance, nitric oxide (NO) production is stimulated in microglia after its S100B protein surrounds the phagosome of opsonized *Cn* in the presence of IFN-γ [17]. *Cn* GXM is also commonly associated with reactive astrocytes [11], which accumulate around cryptococcomas during infection [13]. We recently demonstrated a correlation between glia distribution and GXM localization that varies depending on brain region of infection in mice [12]. Hence, expanding the gap of knowledge on the involvement of glia cells on the immune responses against *Cn* CNS infection is imperative.

IL-6 has pleiotropic effects, including involvement in Ab production, B cell differentiation, T cell activation, and induction of acute phase proteins [18]. IL-6 stimulation of the hypothalamic-pituitary-adrenal axis serves to control the inflammatory response. Thus, IL-6 also has significant indirect anti-inflammatory properties [19]. Elevated levels of IL-6 may have a direct pathogenic role in neurodegenerative diseases such as Alzheimer’s disease and multiple sclerosis [20]. High IL-6 levels are associated with HIV infection [21]. Lack of IL-6 increases blood-brain barrier (BBB) permeability after cryptococcal pulmonary infection [22]. IL-6 is reduced in plasma of individuals with AIDS, and this is related to fungemia and dissemination [23]. Intracerebral challenge of mice with exogenous IL-6 enhances survival during cryptococcal infection by reducing fungal load in blood and brain [24]. Toll-like receptor stimulation increases phagocytosis of *Cn* by microglial cells and cytokine production, including IL-6 [25]. Thus, the role of IL-6 in CNS invasion and colonization, and particularly its impact on glial cells has not been extensively explored.

In this study, we investigated the impact of IL-6 on systemic *Cn* infection in vivo, with emphasis on CNS colonization and glial responses, especially microglia and astrocytes. We compared the survivability and pathophysiology of disseminated cryptococcosis in Wild-type, IL-6^−/−^, and IL-6^−/−^ mice supplemented with exogenous IL-6. We also provided significant evidence demonstrating that IL-6 is an important immunomodulator and influences glial responses and effector functions against cryptococcal infection. In contrast, *Cn* exposure to IL-6 induces capsule enlargement, which may have significant implications in the progression of cryptococcal meningoencephalitis. Our findings provide insight into the dynamic role of IL-6 in neurocryptococcosis and offer novel research avenues in the study of *Cn* pathogenesis.

## Results

### IL-6 deficiency reduces survival in mice systemically infected with *Cn*

**Fig. 1:**
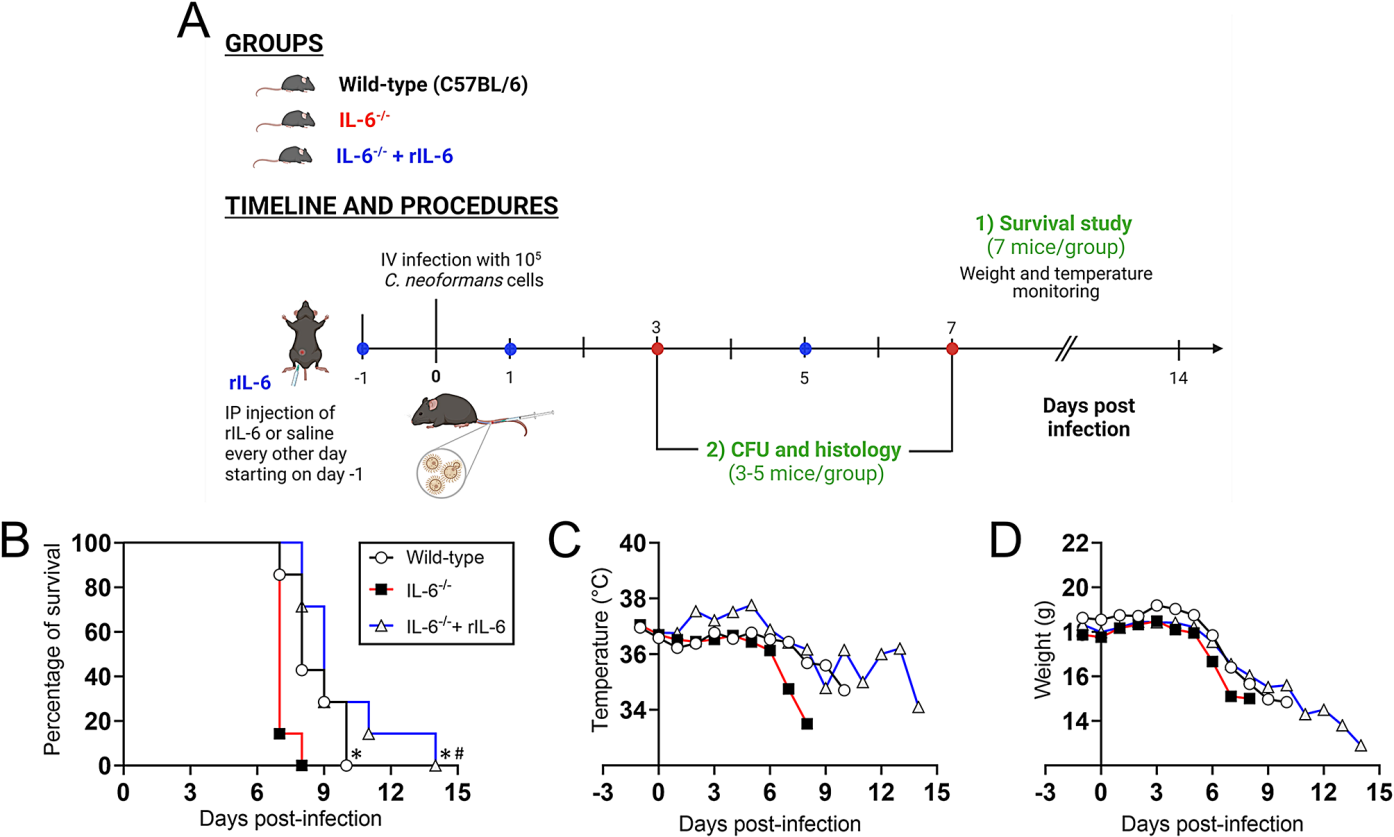
IL-6 deficiency accelerates murine mortality after systemic infection with *Cryptococcus neoformans* (*Cn*). (**A**) Experimental timeline for the systemic (intravenous, IV) *Cn* infection and intraperitoneal (IP) treatment model. Survival and pathological [e.g., colony forming units (CFU) determinations and histology] studies were performed in separate groups of mice. The diagram was created with BioRender.com by Dr. Marta Reguera-Gomez. (**B**) Survival differences (*n* = 7 per group) of C57BL/6 (Wild-type), IL-6^−/−^ (knock-out), and IL-6^−/−^ supplemented IP with 40 pg/g/day of recombinant (r) IL-6 (IL-6^−/−^ + rIL-6) mice IV-infected with 10^5^*Cn* strain H99 cells. Significance (*P* < 0.05) was calculated by log-rank (Mantel-Cox) analysis. Asterisk (*) and hashtag (#) denote higher mortality compared to Wild-type and IL-6^−/−^-infected animals, respectively. (**C**) Temperature and (**D**) body weight were monitored twice daily for changes and development of clinical symptoms indicative of mice nearing humane end points. Each time point corresponds to mean temperature or weight

To evaluate the importance of IL-6 in controlling cryptococcosis, we infected Wild-type C57BL/6, IL-6^−/−^, and IL-6^−/−^ supplemented with recombinant (r) IL-6 (40 pg/g/day) mice with *Cn* strain H99 cells (Fig. 1A). IL-6^−/−^ rodents (median survival: 7-days post infection [dpi]) showed significantly faster mortality than Wild-type- and IL-6^−/−^ + rIL-6-treated mice (median survival: 8- and 9-dpi, respectively; *P* < 0.001; Fig. 1B). In addition, IL-6^−/−^ + rIL-6-treated mice showed prolonged survivability than Wild-type mice (*P* < 0.001; Fig. 1B), with the last animal dying at 14-dpi versus 10-dpi, respectively.

Since IL-6 is involved in temperature regulation in mammals, we monitored changes in core body temperature of Wild-type, IL-6^−/−^, and IL-6^−/−^ + rIL-6 mice after *Cn* infection. Animals in all groups showed a 0.5ºC decrease in their body temperature a day after the rIL-6 pre-infection dose (Fig. 1C). IL-6^−/−^ mice had a considerable temperature drop 6-dpi and correlated with their median mortality. Similarly, Wild-type mice showed a substantial core temperature reduction at 7-dpi. Interestingly, IL-6^−/−^ + rIL-6 mice displayed an approximately 0.5ºC body temperature increase at 2-dpi that was sustained until 5-dpi before fluctuating between 35–36ºC until most animals were dead.

Lastly, we also monitored weight loss in Wild-type, IL-6^−/−^, and IL-6^−/−^ + rIL-6 mice after *Cn* infection as an indicator of disease progression and mortality (Fig. 1D). All the groups showed a similar weight loss trend post-infection indicating that IL-6 deficiency had no impact on body weight loss during *Cn* infection.

Our findings indicate that IL-6 extends mice survival during *Cn* infection and is particularly important in maintaining an optimal temperature.

### IL-6 modulates fungal proliferation during infection

**Fig. 2:**
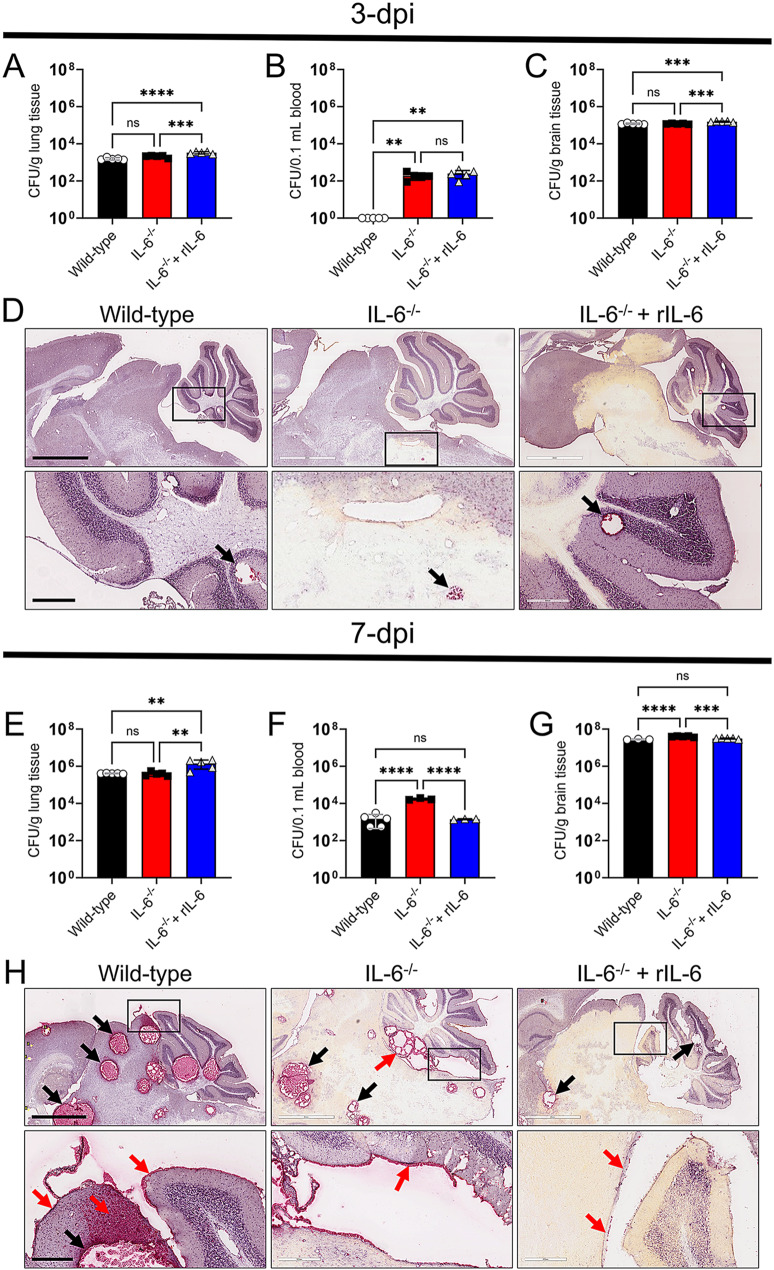
IL-6^−/−^ mice show high cryptococcal burden in circulation and brain 7 days post-systemic infection (dpi). Fungal burden in (**A** and **E**) lung (*n* = 5 mice per group for 3- and 7-dpi), (**B** and **F**) blood (*n* = 5 mice per group for 3-dpi; Wild-type, *n* = 5 mice; IL-6^−/−^ and IL-6^−/−^ + rIL-6, *n* = 3 mice for 7-dpi), and (**C** and **G**) brain (*n* = 5 mice per group for 3-dpi; Wild-type, *n* = 3 mice; IL-6^−/−^ and IL-6^−/−^ + rIL-6, *n* = 5 mice for 7-dpi) collected from Wild-type, IL-6^−/−^, and IL-6^−/−^ + rIL-6 mice IV infected with 10^5^*Cn* H99 cells at 3- and 7-dpi. Quantification of viable yeast cells from infected animals were determined by CFU counting from two dilutions per mouse (*n* = 6 plates per animal) in sterile phosphate-buffered saline (PBS). Each symbol represents a CFU determination per mouse. Bars and error bars denote means and standard deviations (SDs), respectively. Significance (****, *P* < 0.0001; ***, *P* < 0.001; **, *P* < 0.01) was calculated by one-way analysis of variance (ANOVA) and adjusted using Tukey’s post-hoc analysis. ns denotes comparisons which are not statistically significant. Histological examinations of brains removed from *Cn*-infected Wild-type, IL-6^−/−^, and IL-6^−/−^ + rIL-6 mice (*n* = 3 mice per group) at (**D**) 3- and (H) 7-dpi. Uninfected mice were used as tissue baseline controls (not shown). Representative 2X (top panel; scale bar = 2 mm) and 4X (bottom panel; scale bar = 300 μm) magnifications of mucicarmine-stained sagittal sections of the brain are shown. Black rectangular boxes delineate the magnified area (upper to lower panels). Black and red arrows indicate cryptococcomas and mucicarmine (red) staining dissemination, respectively

We investigated the importance of IL-6 in systemic *Cn* disease using colony forming units (CFU) determinations and histopathological examinations of lung and brain tissue removed from *Cn*-infected-Wild-type, -IL-6^−/−^, and -IL-6^−/−^ + rIL-6 mice at 3- and 7-dpi. At 3-dpi, IL-6^−/−^ + rIL-6 mice (lungs, 3.33 × 10^3^ CFU/g; brain, 1.56 × 10^5^ CFU/g) had significantly higher cryptococcal load than Wild-type (lungs, 1.57 × 10^3^ CFU/g; *P* < 0.0001; brain, 1.2 × 10^5^ CFU/g; *P* < 0.001) and IL-6^−/−^ (lungs, 2.1 × 10^3^ CFU/g; *P* < 0.001; brain, 1.2 × 10^5^ CFU/g; *P* < 0.001) mice, respectively (Fig. 2A-C). The lungs and brains of Wild-type- and IL-6^−/−^-infected mice showed no difference in fungal burden. Notably, Wild-type mice showed minimal yeast cells in circulation relative to the IL-6^−/−^ (1.92 × 10^2^ CFU/0.1 mL, *P* < 0.01) and IL-6^−/−^ + rIL-6 (2.5 × 10^2^ CFU/0.1 mL, *P* < 0.01) mice that had similar blood load (Fig. 2B).

Coronal lung tissue sections were stained with hematoxylin-eosin to examine host tissue morphological changes during cryptococcal infection (SFig. 1). Representative pulmonary tissue removed from Wild-type mice (left panels) at 3-dpi displayed localized inflammation (4X) characterized by atelectasis (complete or partial collapse of a lung area) and alveolar emphysema (yellow arrow; 10X). In contrast, in regions of the lung with less inflammation, intrapulmonary bronchioles were also lined by normal epithelium (black arrow; 10X; SFig. 1 A). A high magnification (20X) image exhibited pulmonary emphysematous changes and normal pseudostratified columnar ciliated epithelium lining the bronchioles. IL-6^−/−^ lungs (middle panels) displayed more pronounced inflammation (4X) with considerable atelectasis (yellow arrow), diffuse thickening of interstitial tissue, and intrapulmonary bronchioles with severe hyperplasia of lining epithelium (blue arrow; 10X; SFig. 1 A). High power magnification (20X) of the IL-6^−/−^-infected tissue demonstrated marked thickening of interstitial tissue with severe cell infiltration and severe hyperplasia of the epithelium lining of intrapulmonary bronchiole forming papillary like projections into the lumen (blue arrow). Pulmonary tissue from IL-6^−/−^ + rIL-6 (right panels) evinced more and less inflammation (4X) than that observed in Wild-type and IL-6 deficient lungs, respectively. High magnification images (10-20X) exhibited alveoli with mild thickening of interstitial tissue (yellow arrow; SFig. 1 A) and simple hyperplasia of the epithelium lining the intrapulmonary bronchioles (blue arrow). Remarkably, there was no visible cryptococci nor cryptococcoma formation in the lungs of the animals in any of the groups at this stage (3-dpi) of the infection.

Sagittal brain tissue sections (*n* = 3 mice/group/day) were stained with mucicarmine to identify the morphology of *Cn* in the host tissue during infection (Fig. 2D). Representative 2X brain tissue sections from Wild-type, IL-6^−/−^, and IL-6^−/−^ + rIL-6 mice show minimal differences in fungal colonization at 3-dpi (top panels). Higher magnification (4X; bottom panels) images of Wild-type, IL-6^−/−^, and IL-6^−/−^ + rIL-6 brains displayed small brain cryptococcomas (black arrows).

On day 7 after infection, the lungs of IL-6^−/−^ + rIL-6 mice (1.44 × 10^6^ CFU/g) had significantly higher fungal burden than Wild-type (4.09 × 10^5^ CFU/g; *P* < 0.01) and IL-6^−/−^ (3.92 × 10^5^ CFU/g; *P* < 0.01) mice, respectively (Fig. 2E). However, there were no differences in histopathology in the infected lungs of the compared groups. In fact, lung tissue removed from animals in each experimental group exhibited significant inflammation and cryptococcoma formation (green arrows; 4X; SFig. 1B). They also showed alveoli with atelectasis and thickening of the interalveolar septa (yellow arrows; 10X). Higher magnification of the pulmonary tissue revealed bronchus-associated lymphoid tissue around cryptococcomas in all mice groups (red arrow heads; 20X). Moreover, IL-6^−/−^ (blood, 1.7 × 10^4^ CFU/0.1 mL; brain, 3.96 × 10^7^ CFU/g) mice had a significantly higher fungal load than Wild-type (blood, 1.5 × 10^3^ CFU/0.1 mL, *P* < 0.0001; brain, 2.8 × 10^7^ CFU/g, *P* < 0.0001) and IL-6^−/−^ + rIL-6 (blood, 1.4 × 10^3^ CFU/0.1 mL, *P* < 0.0001; brain, 3.1 × 10^7^ CFU/g, *P* < 0.001) mice in blood (Fig. 2F) and brain (Fig. 2G). There were no differences in fungal load in blood and brain in Wild-type and IL-6^−/−^ + rIL-6 mice.

A representative 2X brain tissue section from a Wild-type mouse euthanized at 7-dpi showed multiple well-circumscribed areas of encephalomalacia or cryptococcomas (black arrows) filled and surrounded with GXM in the cerebral cortex, mid brain, and cerebellum (top left panel; Fig. 2H). A 4X image (bottom left panel) shows the red staining (red arrow in the parenchyma) that extends from cryptococcoma (black arrow) to the cerebral cortex and diffuses to the leptomeninges and subarachnoid space (red arrows). A 2X section from an IL-6^−/−^ mouse displays the mid brain with a well-defined cryptococcoma (black arrows; top middle panel). Additionally, the lumen and walls of the fourth ventricle evinced intense accumulation of mucicarmine staining (red arrow). A high magnification (4X) image demonstrates localized red staining indicative of cryptococci or GXM accumulation in the ependymal and sub-ependymal space lining of the fourth ventricle (red arrow; bottom middle panel). Finally, an IL-6^−/−^ + rIL-6 brain section showed a few cryptococcomas (black arrows) with mucicarmine staining limited to their periphery (top right panel). Unlike the Wild-type and IL-6^−/−^ brains, those supplemented with rIL-6 exhibited minimal mucicarmine staining or cryptococcal accumulation in the ependymal lining of the fourth ventricle (red arrows; bottom right panel).

Overall, our findings indicate that exogenous administration of IL-6 during early (3-dpi) systemic infection increases fungal burden in lungs, blood, and brain. However, as the infection progresses (7-dpi), IL-6^−/−^ mice have higher fungal load in circulation and brain, suggesting the importance of this cytokine in *Cn* systemic dissemination and CNS colonization.

### IL-6^−/−^ mice showed increased *Cn* GXM accumulation in brain tissue

**Fig. 3:**
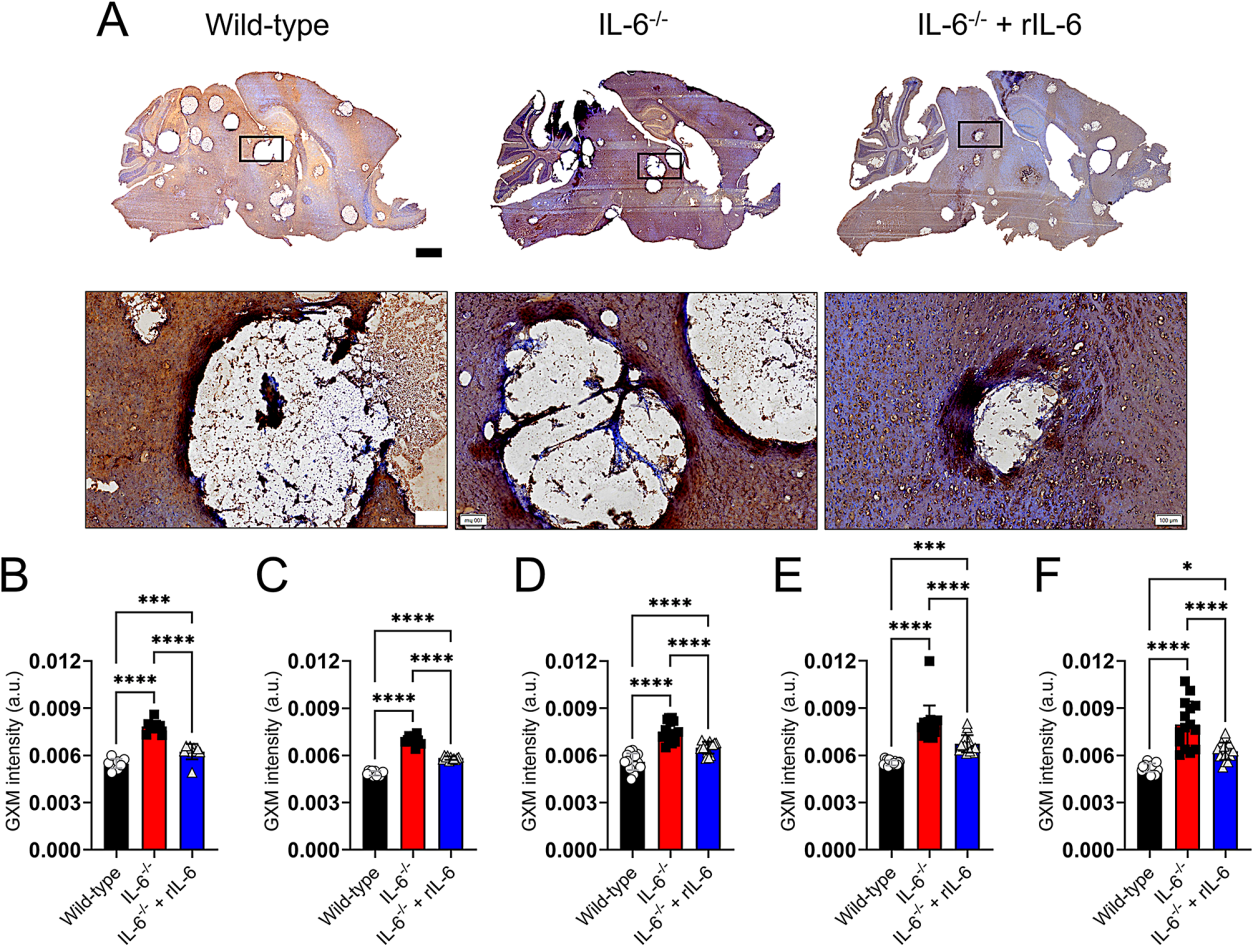
IL-6^−/−^ mice showed significant accumulation of *Cn* glucuronoxylomannan (GXM) in brain tissue. (**A**) Representative images of brain tissue sections (7-dpi) from *Cn* H99-infected Wild-type, IL-6^−/−^ and IL-6^−/−^ + rIL-6 mice (*n* = 3 mice per group) stained with GXM-specific monoclonal antibody (MAb 18B7; brown). Representative 1X (top panel; scale bar: 1 mm) and 4X (bottom panel; scale bar: 100 μm) magnifications are shown. Panel images are a magnification of the black rectangle in the corresponding top-stained section to display tissue morphology surrounding cryptococcoma in each infected group. Quantification of GXM intensity in different brain regions such as (**B**) cortex, (**C**) hippocampus, (**D**) hypothalamus, (**E**) midbrain, and (**F**) cerebellum. Regions of GXM release were evaluated and measured (*n* = 10–15 fields per group; *n* = 3 mice per group) blindly by three independent investigators. Bars and error bars denote means and SDs, respectively. Significance (****, *P* < 0.0001; ***, *P* < 0.001; *, *P* < 0.05) was calculated by one-way ANOVA and adjusted using Tukey’s post-hoc analysis

*Cn* GXM is copiously released during infection causing severe effects to the host immunity. Therefore, we evaluated how IL-6 deficiency impacted GXM accumulation in tissue during cerebral cryptococcosis (Fig. 3). GXM was brown-stained in brain tissue using the specific monoclonal Ab (MAb) 18B7 (Fig. 3A). The GXM intensity in different regions [cortex (Fig. 3B), hippocampus (Fig. 3C), hypothalamus (Fig. 3D), midbrain (Fig. 3E), and cerebellum (Fig. 3F)] of sagittal brain tissue sections was blindly analyzed by three independent investigators (*n* = 10–15 fields per brain region; *n* = 3 mice per group) using NIH ImageJ color deconvolution tool software. Immunolabeling of IL-6^−/−^-infected brain tissue demonstrated extensive GXM accumulation that diffused uniformly throughout the different brain regions (Fig. 3A, middle panels). However, Wild-type mice evinced scattered GXM intensity with most of the polysaccharide accumulating in the mid brain, brainstem, and cerebellum and with lesser extent in the prefrontal cortex (Fig. 3A, left panels). Likewise, the brains of IL-6^−/−^ + rIL-6-infected mice exhibited dispersed GXM localization especially in the brainstem and prefrontal cortex (Fig. 3A, right panels). In all the groups, there was significant GXM deposition surrounding cryptococcomas or areas of encephalomalacia. Analysis of the intensity of GXM staining in brain tissue images from *Cn* Wild-type, IL-6^−/−^, and IL-6^−/−^ + rIL-6-infected mice revealed that animals supplemented with IL-6 had significantly lower GXM intensity or accumulation in all the brain regions analyzed than the IL-6^−/−^-group (*P* < 0.0001; Fig. 3B-F). Moreover, brains removed from IL-6^−/−^ + rIL-6 brains had significantly higher GXM intensity than those excised from Wild-type mice (hippocampus and hypothalamus, *P* < 0.0001; cortex and midbrain, *P* < 0.001; cerebellum, *P* < 0.05; Fig. 3B-F). To validate that Wild-type mice naturally produce IL-6 in the brain during cryptococcal infection and that this cytokine has a crucial role in reducing GXM tissue accumulation, we measured IL-6 levels in homogenates from brains excised at 3- and 7-dpi (SFig. 2). Wild-type-infected mice demonstrated high IL-6 production in brain tissue at 3 (*P* < 0.01)- and 7 (*P* < 0.0001)-dpi relative to Wild-type uninfected mice (*n* = 3 mice/group/day), with a 2-fold increase at 7-dpi over 3-dpi (*P* < 0.001) or as the systemic infection progressed. Hence, our data show that IL-6 reduces fungal capsular polysaccharide brain accumulation, which may have important implications impacting the progression of cerebral cryptococcosis.

### rIL-6 increased *Cn* capsule growth in vitro

**Fig. 4:**
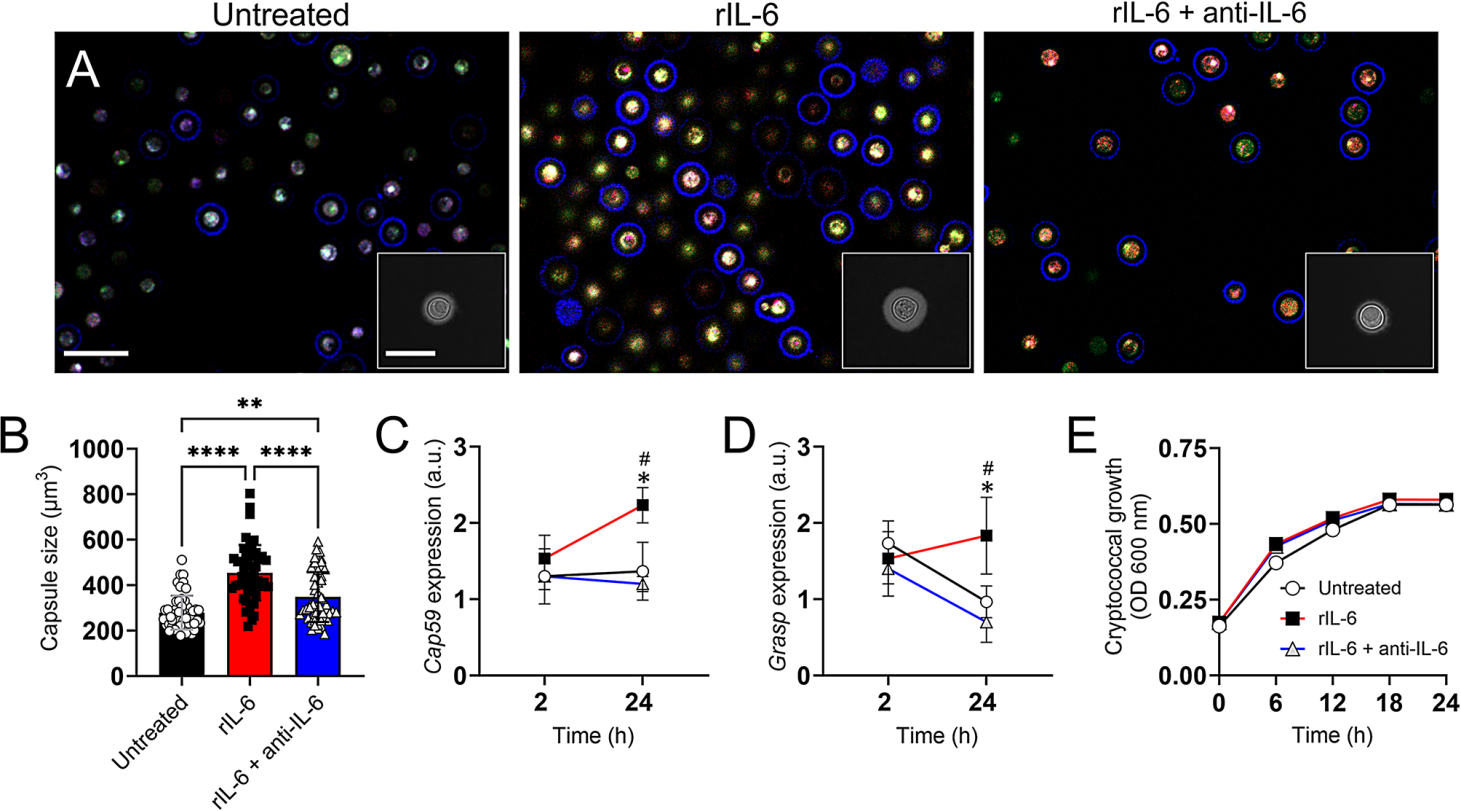
rIL-6 increases *Cn* capsule size. (**A**) Confocal images of *Cn* cells incubated in absence (untreated) and presence of rIL-6 (10 µg/mL) or rIL-6 + anti-IL-6 (20 µg/mL) for 48 h at 37 °C. Immunofluorescent images of cryptococci after incubation with FUN-1 dye (red/green) and MAb 18B7- allophycocyanin-conjugated goat anti-mouse IgG1 (blue) stained to label the cell body (metabolic activity) and capsular polysaccharide (blue), respectively. Insets show India ink-stained fungal cells. Scale bars, field: 25-µm and inset: 5-µm. The pictures were taken at a magnification of 100X. (**B**) Capsule size measurements of untreated and rIL-6- and rIL-6 + anti-IL-6-treated *Cn* cells were performed. The capsule size of 50 cells (each symbol represents 1 cell) per group was measured. Bars and error bars denote means and SDs, respectively. Significance (****, *P* < 0.0001; **, *P* < 0.01) was calculated by one-way ANOVA and adjusted using Tukey’s post-hoc analysis. The relative fold changes of expression (arbitrary units, a.u.) of the capsular-associated genes (**C**) *Cap59* and (**D**) *Grasp* were compared in untreated cryptococci or treated with rIL-6 or rIL-6 + anti-IL-6. The GAPDH gene was used as a reference. Each plotted point represents the means of 3 samples and error bars indicate SDs. Asterisks (*, untreated vs. rIL-6) and hashtags (#, rIL-6 vs. rIL-6 + anti-IL-6) denote *P*-value significance (*P* < 0.05) calculated using two-way ANOVA and adjusted using Tukey’s post-hoc analysis. (**E**) The growth of untreated and rIL-6- or rIL-6 + anti-IL-6-treated cryptococcal cells was monitored in real time using spectrophotometry. A density of 10^6^*Cn* cells were grown in minimal medium alone or supplemented with rIL-6 or rIL-6 + anti-IL-6 at 37 °C under continuous shaking for 24 h in a Bioscreen C plate reader. Optical density (600 nm) measurements were taken at specific time points. Each point represents the average of ten replicates, and error bars denote SDs. The experiments for panels **A**-**E** were performed twice; similar results were obtained each time, and all the results combined are presented

The polysaccharide capsule is the main virulence factor of *Cn*. The extensive secretion of the capsule, and particularly GXM, compromises the immune responses of the host to combat the infection. To confirm our results indicating that IL-6 deficiency promotes *Cn* GXM release in brain tissue, we measured the effect of rIL-6 on capsular size and volume in vitro using immunofluorescence, India ink staining, and light/confocal microscopy (Fig. 4). Specific IL-6 MAb was used to neutralize the effects of this pro-inflammatory cytokine on *Cn* capsular synthesis. Visual inspection of fluorescent images taken from cryptococci cultured in absence (untreated) or presence of rIL-6 (10 µg/mL) or rIL-6 + anti-IL-6 (20 µg/mL) for 48 h at 37 °C revealed that this immunomodulator increases *Cn* capsule volume (blue halos; Fig. 4A). Quantification of India ink-stained microscopic images (Fig. 4A insets; *n* = 50 cells per group) confirmed that treatment with rIL-6 (454.3 µm^3^) significantly stimulated *Cn* capsular volume compared to untreated (279 µm^3^; *P* < 0.0001) and rIL-6 + anti-IL-6 treatment (347.6 µm^3^; *P* < 0.0001; Fig. 4B). Also, rIL-6 + anti-IL-6-exposed cryptococci evinced higher capsule size than untreated fungal cells (*P* < 0.01). Furthermore, to understand how rIL-6 affects *Cn* capsule formation in vitro, we performed quantitative polymerase chain reaction (qPCR) to determine the expression of *Cap59* (Fig. 4C) and *Grasp* (Golgi reassembly and stacking protein; Fig. 4D), which are involved in capsular synthesis [26] and secretion [27], respectively. Culture of *Cn* with rIL-6 for 24 h significantly induced the expression of *Cap59* and *Grasp* relative to untreated (*P* < 0.05 for both) and rIL-6 + anti-IL-6 (*P* < 0.05 for both). Although increasing (2 vs. 24 h; *Cap59*) and decreasing (2 vs. 24 h; untreated and rIL-6 + anti-IL-6; *Grasp*) gene expression trends were observed, these tendencies were not statistically significant (Fig. 4C-D). To assure that the differences in capsule size and related gene expression were not due to variations in fungal growth, we monitored the proliferation of untreated and rIL-6- or rIL-6 + anti-IL-6-treated cryptococci in real time using Bioscreen C analysis (Fig. 4E). Fungal cells in each condition exhibited similar growth for 24 h. Our results demonstrate that IL-6 promotes *Cn* capsule growth, and this effect is independent from cell replication, which may be a potential fungal defense mechanism in response to this host immunomodulator.

### IL-6 deficiency promotes the presence of dystrophic microglia during *Cn* infection

**Fig. 5:**
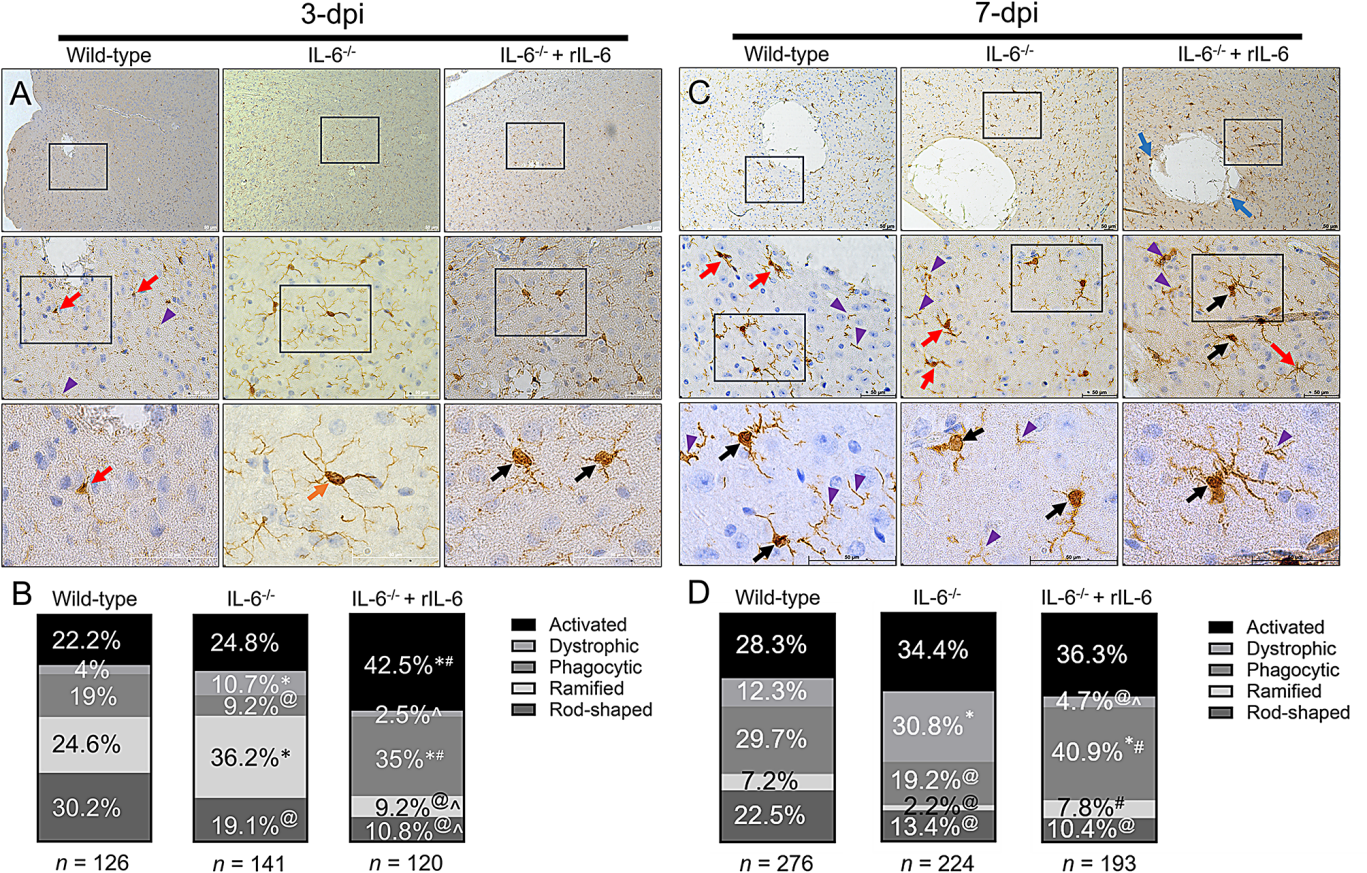
*Cn*-infected IL-6^−/−^ brains have a high number of dystrophic microglia at 7-dpi. Microglial responses to *Cn* H99 infection in brain tissue excised from Wild-type, IL-6^−/−^, and IL-6^−/−^ + rIL-6 mice (*n* = 3 mice per group) at (**A**) 3- and (**C**) 7-dpi. Uninfected mice were used as tissue baseline controls (not shown). Representative 10X (top panel; scale bar = 200 μm), 40X (central panel; scale bar = 50 μm), and 100X (bottom panel; scale bar = 50 μm) magnifications of ionized calcium-binding adaptor protein (Iba-1)-binding MAb-stained sections of the brain are shown. Black rectangular boxes delineate the area magnified (top to bottom panels). Brown staining indicates microglia. Orange, black, red, and blue arrows indicate ramified or branched, hypertrophic or activated, dystrophic, and phagocytic or amoeboid microglia, respectively. Purple arrow heads denote microglial ramification or branch debris. The percentage of microglial phenotype abundance in brain tissue of Wild-type (3-dpi, *n* = 126 cells; 7-dpi, *n* = 276 cells), IL-6^−/−^ (3-dpi, *n* = 141 cells; 7-dpi, *n* = 224 cells), and IL-6^−/−^ + rIL-6 (3-dpi, *n* = 120 cells; 7-dpi, *n* = 193 cells) mice at (**B**) 3- and (**D**) 7-dpi. Microglia were classified according to their morphology as activated, dystrophic, phagocytic, ramified, or rod-shaped. Brain regions were also evaluated and measured (*n* = 12 fields per group) blindly by three independent investigators. Significance (*, *P* < 0.05) was calculated by one-way ANOVA and adjusted using Tukey’s post-hoc analysis. The asterisk (*) and hashtag (#) symbols indicate higher percentage than Wild-type and IL-6^−/−^ groups, respectively. The at sign (@) and wedge (^) symbols indicate lower percentage than Wild-type and IL-6^−/−^, respectively

Microglia are the resident phagocytes of the CNS, involved in surveillance and immune recruitment, and important inducers of inflammatory responses through cytokine signaling. We recently described that *Cn* infection modifies microglial morphology from branched or ramified (cylindrical soma with long and thin ramifications) to either activated or hypertrophic (thick soma and thick ramifications), phagocytic or amoeboid (large soma and short retracted ramifications; ameboid or macrophage-like), dystrophic (not-well defined soma and thin ramifications), or rod-shaped (enlarged cylindrical soma and polar ramifications) phenotypes, which may influence the progression and outcome of cerebral cryptococcosis [28]. IL-6 has been shown to stimulate brain repair by microglia particularly in traumatic brain injury [29]. Hence, we examined how IL-6 deficiency alters the morphology of microglia during cryptococcal infection at 3- and 7-dpi (Fig. 5). Microglia were immunolabeled using ionized calcium-binding adaptor molecule-1 (Iba-1; brown)-binding MAb (Fig. 5A and C). Images of each group treatment were visualized under the microscope (Fig. 5A and C) and the morphology of microglia (Fig. 5B and D) was qualitatively documented as previously described [28, 30]. Microglia were classified as activated or hypertrophic, dystrophic, phagocytic, or amoeboid, ramified, and rod-shaped [28].

At 3-dpi, the brain of a Wild-type mouse (10X, top left panel) shows a cryptococcal lesion surrounded by Iba-1^+^ cells or microglia (Fig. 5A). High magnification (40X, center and 100X, bottom left panels) of Wild-type tissue demonstrate dystrophic microglia (red arrows) near the area of encephalomalacia consisting of a small cell body with a distorted surface and loss of their processes (purple arrowheads). IL-6^−/−^-infected brain tissue displayed ramified microglia (40X, center middle panel) which is characterized by having a relatively small ovoid cell body surrounded by very thin branches (orange arrow; 100X, bottom middle panel). The brain of an IL-6^−/−^ + rIL-6-infected mouse exhibited increased microgliosis or increased number of microglia (10X, upper right panel). High magnification of IL-6^−/−^ + rIL-6 brain tissue showed a cryptococcal lesion surrounded by activated and phagocytic microglia (black arrows; 40X, middle and 100X, bottom right panels).

Brain sections of IL-6^−/−^ + rIL-6 had a significantly higher percentage of activated (42.5%) and phagocytic (35%) microglia than tissue from Wild-type (22.2%, activated; 19% phagocytic; *P* < 0.05) and IL-6^−/−^ (24.8%, activated; 9.2% phagocytic; *P* < 0.05) animals (Fig. 5B). IL-6^−/−^-infected brain tissue exhibited a significantly higher percentage of dystrophic (10.7%) microglia compared to Wild-type (4%; *P* < 0.05) and IL-6^−/−^ + rIL-6 (2.5%; *P* < 0.05) mice. Likewise, brain tissue from IL-6^−/−^-infected mice showed a significantly higher percentage of branched or ramified (36.2%) microglia relative to the Wild-type (24.6%; *P* < 0.05) and IL-6^−/−^ + rIL-6 (9.2%; *P* < 0.05) groups. Wild-type brains evinced a significantly higher percentage of ramified microglia than IL-6^−/−^ mice (*P* < 0.05). Lastly, rod-shaped (30.2%) microglia were the most abundant phenotype in infected Wild-type brain tissue at 3-dpi, with a higher percentage of this morphology relative to brain tissue from IL-6^−/−^ (19.1%; *P* < 0.05) and IL-6^−/−^ + rIL-6 (10.8%; *P* < 0.05). The percentage of rod-shaped microglia in IL-6^−/−^ tissue was also significantly higher than in IL-6^−/−^ + rIL-6 tissue (*P* < 0.05).

The brains of Wild-type (10X, top left panel) and IL-6^−/−^ + rIL-6 (10X, top right panel) mice excised at 7-dpi displayed increased microgliosis around the cryptococcomas (Fig. 5C). In contrast, IL-6^−/−^ brains exhibited less microglia surrounding the area of encephalomalacia (10X, top middle panel). Higher magnification of Wild-type brain tissue evinced dystrophic glial cells near the cryptococcoma (red arrows; 40X, center left panel) with close activated or hypertrophic microglia (black arrows; 40X, center and 100X, bottom left panels). IL-6^−/−^ brains show dystrophic microglia (red arrows; 40X, center middle panel) and activated microglia that appears to be dropping their ramifications to become phagocytic (black arrows; 100X, center bottom panel). IL-6^−/−^ + rIL-6 brain tissue had considerable recruitment of phagocytic (blue arrows; 10X; top right panel) and activated (black arrows; 40X, right middle and 100X, bottom panels) microglia near the cryptococcal brain lesion. Notably, brain tissue from all the groups exhibited accumulation of microglial ramification or branch debris (purple arrow heads) that may have significant implications in *Cn* infection control.

IL-6^−/−^ + rIL-6 brains had larger percentage of phagocytic microglia (40.9%) than Wild-type (29.7%; *P* < 0.05) and IL-6^−/−^ (19.2%; *P* < 0.05) brains at 7-dpi (Fig. 5D). In contrast, the brains of IL-6^−/−^ mice showed a higher percentage of dystrophic cells (30.8%) than Wild-type (12.3%; *P* < 0.05) and IL-6^−/−^ + rIL-6 (4.7%; *P* < 0.05) groups. All the groups have proportionally similar activated or hypertrophic microglia. Wild-type brains have the largest percentage of rod-shaped (22.5%; *P* < 0.05) cells. Branched or ramified microglia was the least observed phenotype found in the brains of the compared groups, although Wild-type (7.2%) and IL-6^−/−^ + rIL-6 (7.8%) mice had similar percentage of this morphology, whereas IL-6^−/−^ mice had the lowest percentage (2.2%; *P* < 0.05).

Taken together, our in vivo data suggest that IL-6 prevents brain colonization, potentially through its effects on microglia morphology and their effector functions.

### NR-9460 microglia-like cells treated with IL-6 small interfering RNA (siRNA) exhibited decreased cryptococcal phagocytosis

**Fig. 6:**
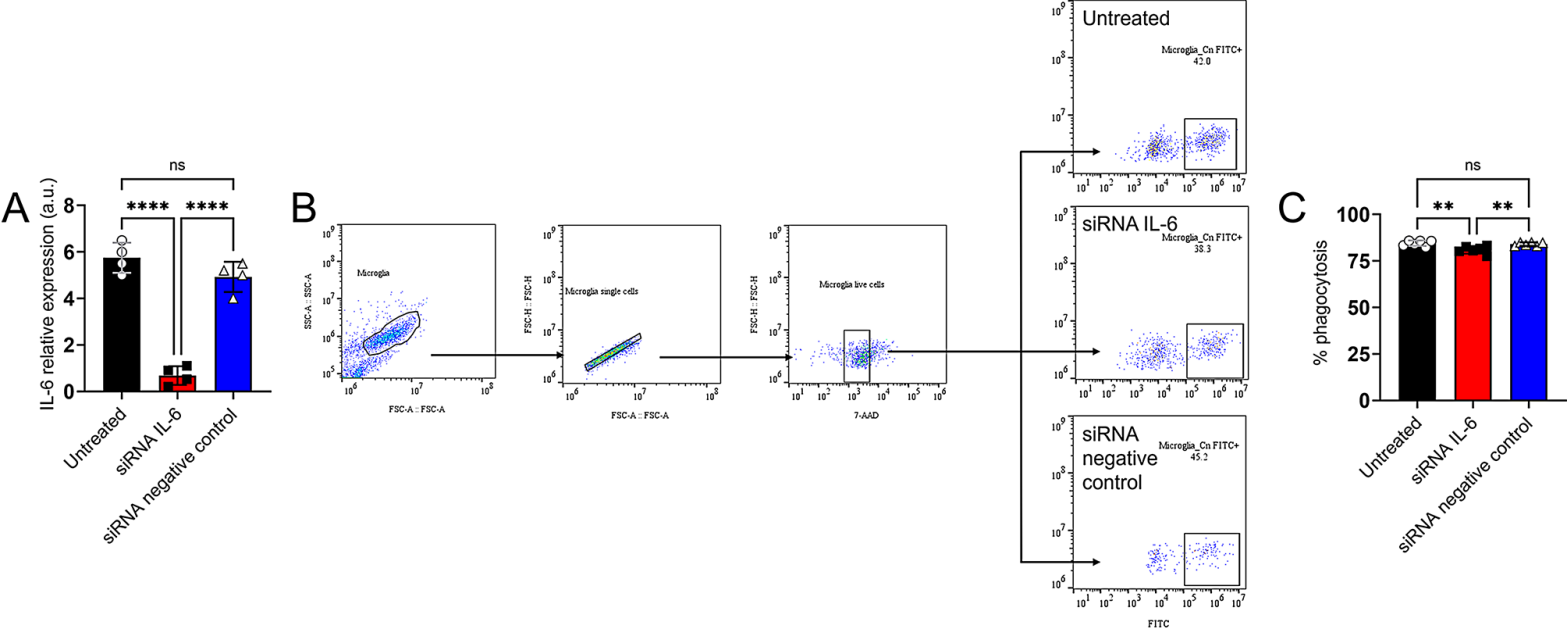
Small interfering RNA (siRNA) IL-6-treated NR-9460 microglia-like cells demonstrated reduced phagocytosis of *Cn*. (**A**) The relative fold changes of expression of the IL-6 gene were compared in untreated, siRNA IL-6, and siRNA negative control transfected NR-9460 cells (*n* = 4 replicates per group) after 2 h activation. (**B**) Representative flow cytometry dot plots show the gating strategy followed to define phagocytic microglia. The gating strategy separated the cells based on forward (FSC) and side scatter (SSC) in order to remove cell aggregates, small debris, or cryptococci followed by single-cell gating (FSC-A and FSC-H). Next, live cells were defined as a 7-aminoactinomycin D (7-AAD)-negative population (dead cells were excluded), and these live cells were further analyzed for FITC fluorescence. FITC-positive yeasts were used to establish the fluorescent threshold for phagocytic microglia. Live single-cell and FITC-positive microglia were defined as phagocytic microglia. Representative dot plots for untreated, siRNA IL-6 and siRNA negative control transfected NR-9460 cells incubated with cryptococci are shown. (**C**) The percentage of phagocytosis was determined by flow cytometry after 4 h incubation of 5 × 10^5^ NR-9460 cells with FITC-conjugated MAb 18B7 (IgG_1_)- 5 × 10^6^ opsonized *Cn* strain H99 cells (1 microglia: 10 fungi ratio). Each symbol represents an independent replicate (*n* = 6) where ≥ 10,000 events per group were measured. For **A** and **C**, incubations were performed at 37 °C and 5% CO_2_. Bars and error bars denote means and SDs, respectively. Significance (****, *P* < 0.0001; **, *P* < 0.01) was calculated by one-way ANOVA and adjusted using Tukey’s post-hoc analysis. ns denotes comparisons which are not statistically significant

Phagocytosis is one of the main activities associated with microglial responses to infection. Therefore, we assessed the impact of IL-6 on fungal phagocytosis by NR-9460 microglia-like cells. For that, we first transfected NR-9460 cells with IL-6 siRNA for 24 h to silence this cytokine gene and inhibit protein production. We similarly treated NR-9460 cells with a mock siRNA molecule and used these cells as a negative control. To ensure that siRNA treatments did not affect the viability of NR-9460 microglia-like cells, we performed flow cytometry analysis (*n* = 4 replicates per group) and compared siRNA groups to untreated cells (SFig. 3). Representative flow cytometry dot plots are shown (SFig. 3 A). On average, untreated (91.9%), siRNA IL-6 (88.8%), and siRNA negative control (84.4%) NR-9460 cells show similar cell viability percentage. Then, we activated untreated, siRNA IL-6, and siRNA negative control NR-9460 cells with bacterial lipopolysaccharide (LPS; 0.5 µg/mL)/IFN-γ (5 ng/mL) in Opti-MEM I medium with 2% fetal bovine serum (FBS) for 2 h and collected their culture supernatant after 24 h incubation at 37 °C and 5% CO_2_ (Fig. 6A). Transfected cells with siRNA IL-6 evinced a significant 9 to 10-fold reduction in IL-6 production compared to untreated (*P* < 0.0001) and siRNA negative control (*P* < 0.0001) cells. There was no difference in IL-6 production between untreated and siRNA negative control cells. We showed the flow cytometry gating strategy utilized to quantify cryptococcal phagocytosis and representative dot plots for each condition (Fig. 6B). siRNA IL-6 (81%)-treated NR-9460 microglia-like cells had significantly lower fungal phagocytosis percentage than untreated (84.5%; *P* < 0.01) and siRNA negative control (84.1%; *P* < 0.01) cells (Fig. 6C). Our findings suggest that IL-6 production by microglia, although marginally, enhances cryptococcal phagocytosis, which is important for infection control especially upon brain penetration.

### rIL-6 enhances cryptococcal phagocytosis and killing by microglia in vitro

**Fig. 7:**
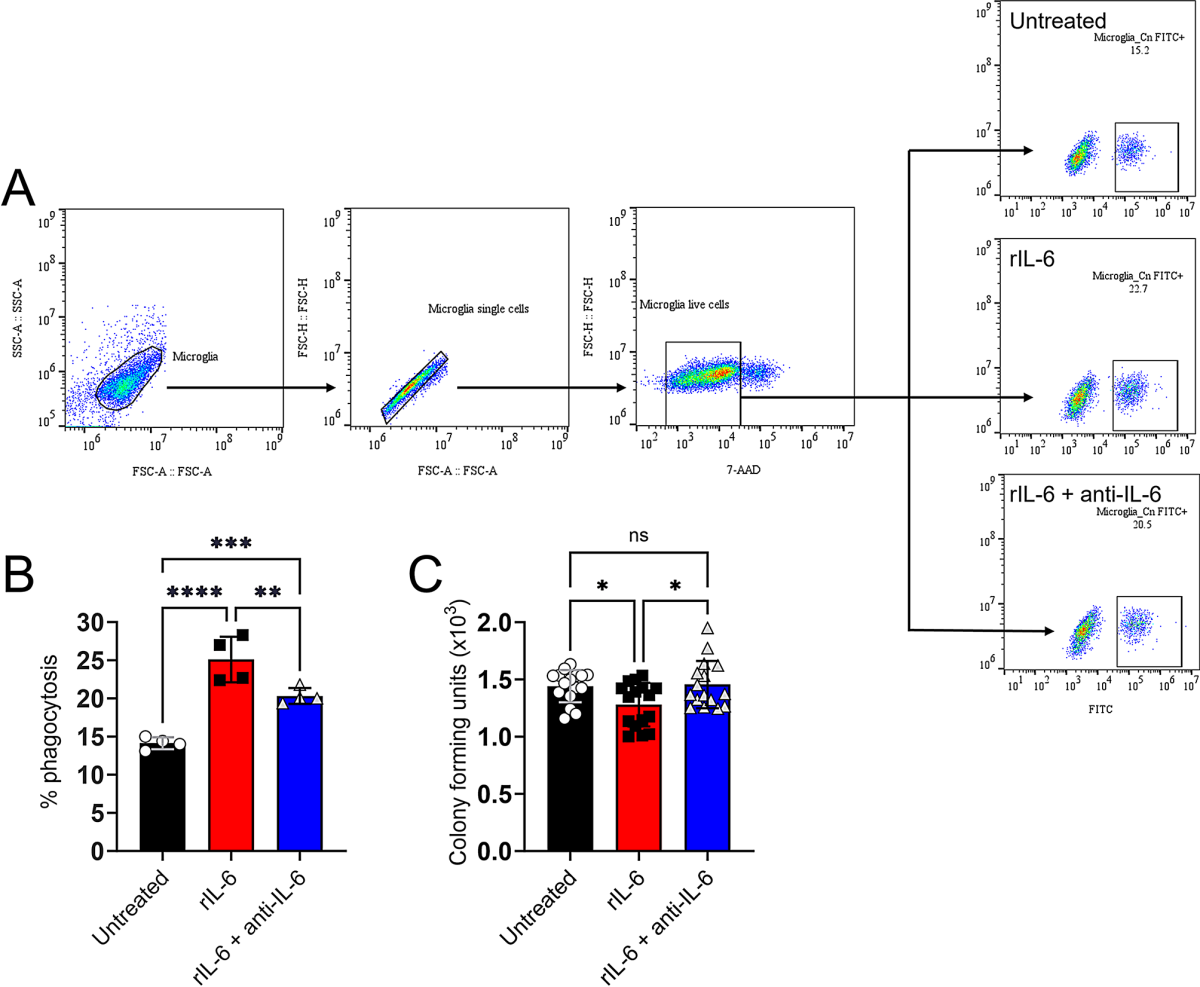
rIL-6 promotes phagocytosis and killing of *Cn* by NR-9460 microglia-like cells. (**A**) Representative flow cytometry dot plots for untreated and rIL-6- or rIL-6 + anti-IL-6-treated NR-9460 cells incubated with cryptococci are shown. (**B**) The percentage of phagocytosis was determined by flow cytometry after 4 h incubation of 5 × 10^5^ NR-9460 cells with FITC-conjugated MAb 18B7-opsonized 5 × 10^6^*Cn* strain H99 cells (1 microglia: 10 fungi ratio). Each symbol represents an independent replicate (*n* = 4) where ≥ 10,000 events per group were measured. (**C**) The number of *Cn* H99 cells killed by microglia-like cells was determined by CFU after 24 h incubation. Each symbol represents an individual CFU count (*n* = 16 per group). For **B** and **C**, incubations were performed at 37 °C and 5% CO_2_. Bars and error bars denote means and SDs, respectively. Significance (****, *P* < 0.0001; ***, *P* < 0.001; **, *P* < 0.01; *, *P* < 0.05) was calculated by one-way ANOVA and adjusted using Tukey’s post-hoc analysis. ns denotes comparisons which are not statistically significant

We investigated the role of rIL-6 on the efficacy of GXM-specific MAb 18B7-mediated phagocytosis and killing of *Cn* by NR-9460 microglia-like cells after 4 h at 37 °C and 5% CO_2_ (Fig. 7). The gating strategy used in the flow cytometry analysis to quantify cryptococcal phagocytosis by NR-9460 cells as well as the representative dot plots for each experimental condition are shown (Fig. 7A). NR-9460 cells cultured with rIL-6 demonstrated higher phagocytosis of cryptococci (25.1%) compared to untreated (14.13%; *P* < 0.0001) and rIL-6 + anti-IL-6 (20.3%; *P* < 0.01) microglia-like cells (Fig. 7B). rIL-6 + anti-IL-6-treated microglia phagocytized more fungal cells than the untreated counterparts (*P* < 0.001). In addition, microglial cells incubated with rIL-6 evinced higher cryptococcal killing than untreated (*P* < 0.05) and rIL-6 + anti-IL-6-treated cells (*P* < 0.05; Fig. 7C). There was no difference in *Cn* killing between untreated and rIL-6 + anti-IL-6-treated microglia. These results indicate that IL-6 stimulates microglia to engulf and kill *Cn* cells, thus impacting the capacity of these CNS myeloid cells to control the infection and disease progression.

### *Cn* infection induces only minor astrocytic responses in IL-6^−/−^ mice

**Fig. 8:**
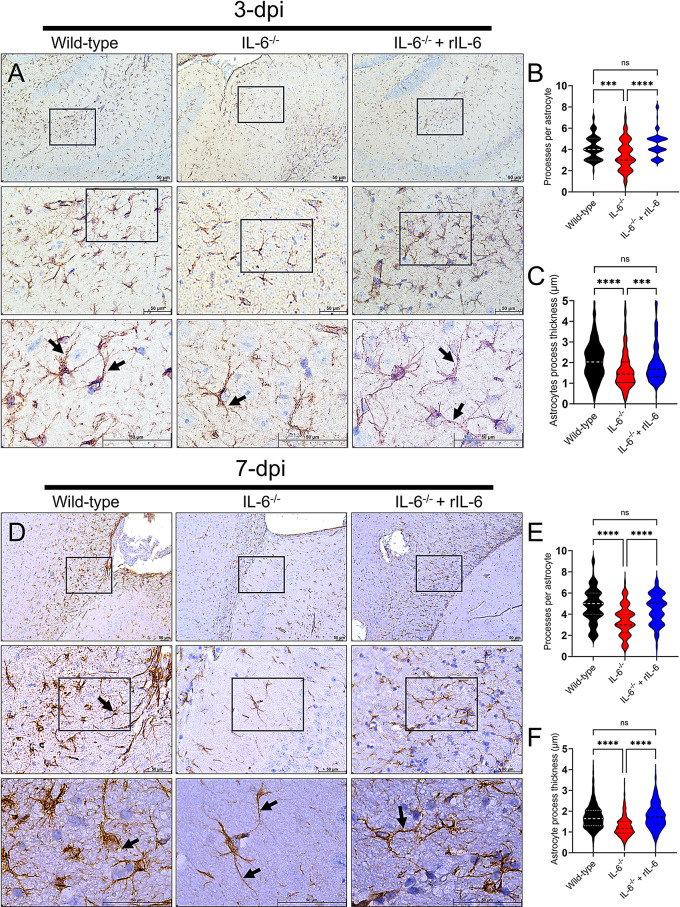
*Cn* infection induces only minor astrocytic responses in the hippocampus of IL-6^−/−^ mice. Histological examinations of the hippocampus from brains removed from *Cn*-infected Wild-type, IL-6^−/−^, and IL-6^−/−^ + rIL-6 mice (*n* = 3 mice per group) at (**A**) 3- and (**D**) 7-dpi. Uninfected mice were used as tissue baseline controls (not shown). Representative 10X (top panel; scale bar = 200 μm), 40X (central panel; scale bar = 50 μm), and 100X (bottom panel; scale bar = 50 μm) magnifications of glial fibrillary acid protein (GFAP)-binding MAb-stained sections of the brain are shown. Black rectangular boxes delineate the area magnified (top to bottom panels). Brown staining indicates astrocytes. Black arrows indicate astrocytic processes. Processes per astrocyte [(**B**) 3-dpi: Wild-type, *n* = 105 cells; IL-6^−/−^, *n* = 93 cells; IL-6^−/−^ + rIL-6, *n* = 103 cells; (**E**) 7-dpi: Wild-type, *n* = 91 cells; IL-6^−/−^, *n* = 92 cells; IL-6^−/−^ + rIL-6, *n* = 71 cells] and astrocyte process thickness (in µm; (**C**) 3-dpi: Wild-type, *n* = 284 processes; IL-6^−/−^, *n* = 305 processes; IL-6^−/−^ + rIL-6, *n* = 290 processes; (**F**) 7-dpi: Wild-type, *n* = 326 processes; IL-6^−/−^, *n* = 355 processes; IL-6^−/−^ + rIL-6, *n* = 352 processes) were determined in hippocampal tissue sections using an inverted microscope. For **B**-**C** and **E-F**, violin plots denote the means (dashed lines) and replicate distributions. Asterisks denote significance (****, *P* < 0.0001; ***, *P* < 0.001) as calculated by one-way ANOVA and adjusted using Tukey’s post-hoc analysis. ns denotes comparisons which are not statistically significant

*Cn* causes astrocytes to become reactive during infection [13, 14] and these morphological alterations probably have a critical role in cryptococcal CNS colonization and meningoencephalitis development. Therefore, we assessed the astrocytic responses and morphological changes in brain tissue of Wild-type, -IL-6^−/−^, and -IL-6^−/−^ + rIL-6 following systemic cryptococcal infection at 3- and 7-dpi (Fig. 8). Astrocytes were immunolabeled with glial fibrillary acidic protein (GFAP; brown)-binding MAb. At 3-dpi, images of Wild-type brains exhibited astrocytosis with protoplasmic astroglia surrounding neurons and fibrous astrocytic subtypes surrounding the wall of blood capillaries in the white matter (10X, top left panel; Fig. 8A). High magnification images (40 and 100X) of Wild-type brains displayed astrogliosis in the hippocampal parenchyma. Low (10X, center top panel) and high (40-100X, center middle and bottom panels) magnification images of IL-6^−/−^ hippocampal tissue showed normal astrocytes with thin processes and without any reactive morphological changes. Finally, the hippocampus of IL-6^−/−^ + rIL-6 mice exhibited considerable astrocytosis and astrogliosis (10-40X, top and middle right panels; Fig. 8A). High magnification images (100X, bottom right panel) of IL-6^−/−^ + rIL-6 hippocampal tissue displayed astrocytes with long thick-branched processes forming an arborizing pattern around neurons and blood vessels. To quantify the morphology of astrocytes in brains excised from Wild-type, IL-6^−/−^, and IL-6^−/−^ + rIL-6-infected mice at 3-dpi, we used light microscopy to measure their processes number and thickness (Fig. 8B-C). Wild-type- and IL-6^−/−^ + rIL-6-astrocytes displayed significantly higher number of processes (*P* < 0.001 and *P* < 0.0001, respectively; Fig. 8B) and thicker processes (*P* < 0.0001 and *P* < 0.001, respectively; Fig. 8C) relative to IL-6^−/−^-derived astrocytes. No differences in astrocyte morphology were observed between Wild-type- and IL-6^−/−^ + rIL-6 groups.

At 7-dpi, images of Wild-type brains displayed an increased number of brown-stained protoplasmic astrocytes in the hippocampus (10X, top left panel; Fig. 8D). Interestingly, astrogliosis was observed (black arrow; 40X, center left panel) and the processes of an astrocyte were shown surrounding a blood vessel (black arrow; 100X, bottom left panel). IL-6^−/−^ hippocampal tissue demonstrates minimal astrogliosis (10X, top middle panel) and long and thin astrocytic processes (black arrows; 40-100X; Fig. 8D). Lastly, the hippocampus of IL-6^−/−^ + rIL-6 exhibited moderate astrocytosis and astrogliosis (10X, top right panel; Fig. 8D) with accumulation of astrocytes with intermediate process thickness (100X, bottom right panel). We also quantified the morphology of astrocytes in brains excised from Wild-type, IL-6^−/−^, and IL-6^−/−^ + rIL-6-infected mice at 7-dpi (Fig. 8E-F). Wild-type- and IL-6^−/−^ + rIL-6-astrocytes displayed significantly higher number of processes (*P* < 0.0001 for both; Fig. 8E) and thicker processes (*P* < 0.0001 for both; Fig. 8F) relative to IL-6^−/−^-derived astrocytes. No differences in astroglial morphology were observed between Wild-type- and IL-6^−/−^ + rIL-6 groups.

These findings show that brains removed from IL-6^−/−^ mice have a weaker astrocytic response than Wild-type- or IL-6^−/−^ + rIL-6 and that this response impacts considerably the ability of the immune response to control cryptococcal CNS infection and clearance.

## Discussion

Using an IL-6 knock-out (KO) mouse model of systemic *Cn* infection, we demonstrated and validated the importance of this multifunctional cytokine on disseminated cryptococcosis, particularly focusing on fungal brain invasion and colonization. However, mice deficient in IL-6 had more pulmonary inflammation than Wild-type and IL-6^−/−^ + rIL-6 mice at 3-dpi even when IL-6^−/−^ + rIL-6 lungs had higher fungal load than those from Wild-type and IL-6^−/−^ mice. Since Wild-type mice actively produce IL-6 early during cryptococcal infection, these animals were able to limit the inflammatory response to localized regions of the lungs while keeping the fungal burden lower than IL-6^−/−^ and IL-6^−/−^ + rIL-6 groups. As the infection progressed, mice deficient in IL-6 had similar histological respiratory disease development compared to animals with intact IL-6 responses or supplemented with the exogenous cytokine. The lungs of mice in each experimental group displayed considerable inflammation, similar cryptococcoma formation, and comparable fungal burden at 7-dpi, suggesting that IL-6 production may only play a critical role early in cryptococcal pulmonary disease, or its function can be substituted by other cytokines involved in the acute phase response such as IL-1β and TNF-α.

IL-6^−/−^ mice exhibited faster mortality than animals that naturally produced or were supplemented with the cytokine likely due to their inability to control fungal replication in circulation or penetration into the CNS. In this regard, IL-6 has been shown to contribute to the inhibition of cryptococcal proliferation [31], which may explain the reduced fungal load in the blood of Wild-type and IL-6^−/−^ + rIL-6 mice. Also, IL-6^−/−^ mice have been shown to increase sepsis severity and mortality in a model of cecal ligation and puncture [32], which may explain why IL-6^−/−^ + rIL-6 mice evinced comparable high number of cryptococci in blood to IL-6^−/−^ animals at 3-dpi. Nevertheless, consistent rIL-6 administration to IL-6-deficient mice controlled the fungal load in circulation to equivalent numbers to those observed in Wild-type mice at 7-dpi. Low IL-6 levels in CSF are correlated with reduced survival in patients with HIV/AIDS and cryptococcal meningoencephalitis [33]. IL-6 deficiency has been previously shown to compromise the BBB integrity [22], facilitating *Cn* brain invasion, which explains the increased fungal burden in these mice compared to those with IL-6. IL-6 neutralization after specific MAb administration to C57BL/6 mice has been shown to increase the BBB permeability of *Cn* [22]. We also confirmed that injection of IL-6 to KO mice reduces cryptococcal invasion into the CNS likely by strengthening the integrity of the BBB [22]. Moreover, resistance to *Cn* in the brains of mice correlated with the local production of IL-6 and IL-1β, and that resistance increases by the addition of either one exogenously before fungal challenge [24]. These previous observations also support our findings showing that IL-6^−/−^ brains had higher fungal burden than Wild-type and IL-6^−/−^ + rIL-6 mice.

Brain tissue removed from IL-6^−/−^ mice had substantial accumulation of *Cn* GXM. GXM aggregation reduces inflammation [33] and prevents immune cell infiltration into the brain during disseminated infection [34]. We recently demonstrated that GXM disrupts endothelial cell tight junctions in the BBB, facilitating the transmigration of *Cn* into the CNS [3]. In contrast, Wild-type mice showed significantly lower GXM intensity in the brain than IL-6^−/−^ and IL-6^−/−^ + rIL-6 mice. To rule out that the reduction of GXM accumulation observed in Wild-type mice was not attributed to being unable to actively producing IL-6 in infected brain tissue, we compared uninfected vs. infected Wild-type mice and confirmed that Wild-type-infected mice produced IL-6 in brain tissue and that its concentrations increased as the infection progressed. Beenhouwer et al., previously demonstrated that Wild-type mice had lower GXM in serum than IL-6 KO mice [35]. Although we did not measure the levels of GXM in circulation (e.g., serum or CSF), high GXM levels in the blood of HIV/AIDS patients with cryptococcosis are associated with lower IgG production and mortality [36]. Secreted GXM accumulates in patient serum and CSF at µg/mL concentrations and has well-documented immunosuppressive properties, correlating with poor patient outcomes. For example, we recently demonstrated in intracerebrally infected mice that mortality was associated with the presence of subarachnoid hemorrhaging and GXM deposition in the meningeal blood vessels and meninges in all brain regions infected [12]. Wild-type and IL-6^−/−^ + rIL-6 brains exhibited lower GXM intensity, validating that IL-6 is critical and beneficial for mouse survival by attenuating the progression of neurocryptococcosis [24]. Importantly, the administration of rIL-6 is artificial, compared to what occurs in infected Wild-type mice, thus, explaining the higher GXM tissue accumulation observed in IL-6^−/−^ + rIL-6 mice and vice versa when compared to IL-6^−/−^ mice.

**Fig. 9:**
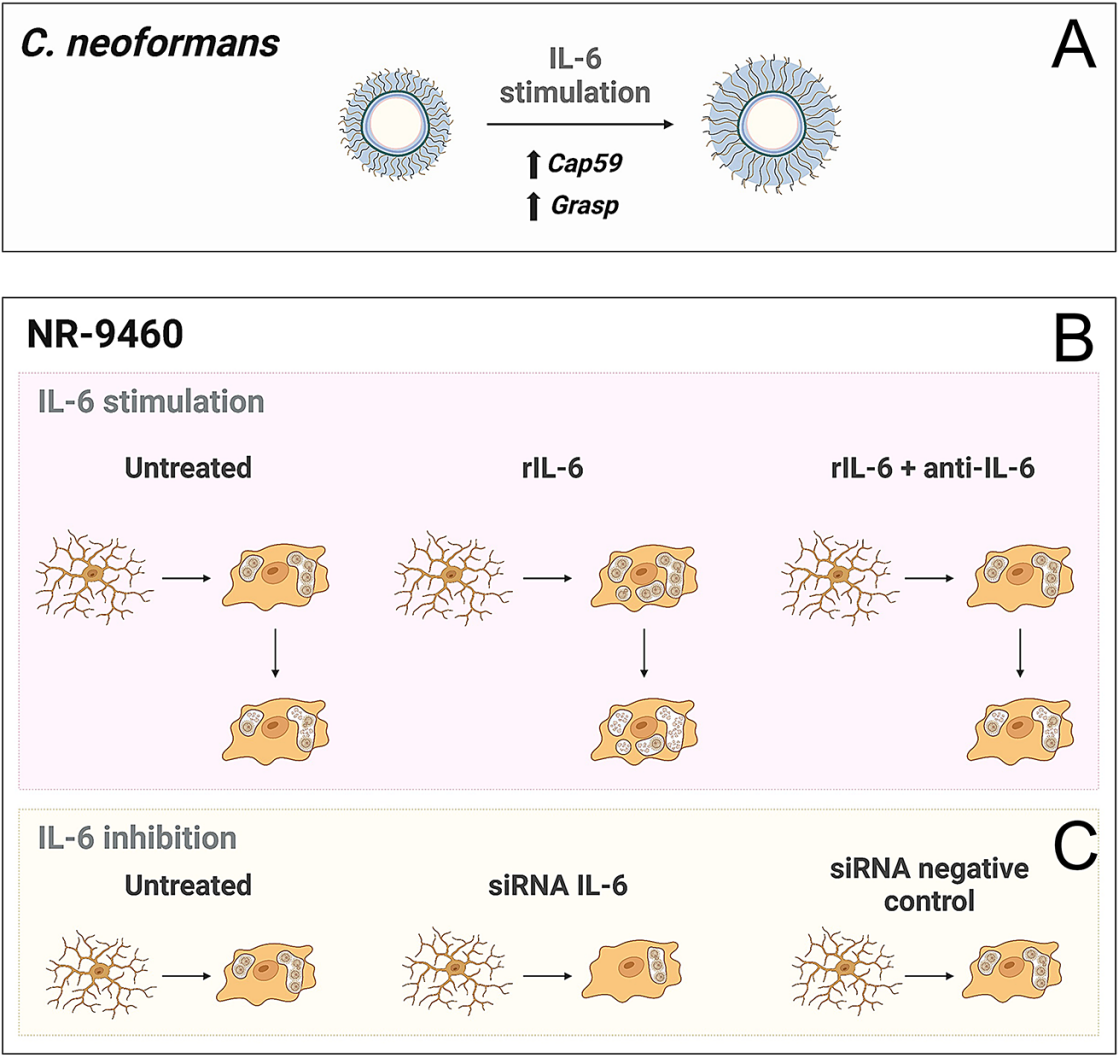
Effect of IL-6 on NR-9460 microglia-like cells and *Cn* cells in vitro. (**A**) Exposure of cryptococci to rIL-6 stimulates capsular-related gene expression (e.g., *Cap59* and *Grasp*) and promotes enlargement of its polysaccharide capsule. (**B**) The stimulation of microglia by rIL-6 promotes phagocytosis and killing of *Cn*. (**C**) The inhibition of IL-6 via siRNA transfection results in reduced phagocytosis. Each individual model was created with BioRender.com by Dr. Marta Reguera-Gomez

*Cn* can adaptively modulate the size and polysaccharide release of its capsule according to external stimuli, which can be advantageous to the fungus during pathogenesis. Exposure of cryptococci to rIL-6 induced the expression of capsular-related genes *Cap59* and *Grasp* and significantly stimulated the enlargement of the capsule (Fig. 9A) relative to untreated and IL-6 + anti-IL-6 cells. Also, rIL-6-mediated capsule size augmentation by *Cn* was independent of fungal proliferation since cryptococci grew similarly in each condition in vitro. Activation of peripheral blood mononuclear cells after interactions with *Cn* increases the levels of IL-6 and augments their resistance to infection [31]. Clinical cryptococcal strains producing larger ex vivo capsules in the baseline CSF correlated with higher intracranial pressure, slower fungal clearance, and paucity of CSF inflammation, including decreased CSF white blood cell count, IL-4, IL-6, IL-7, IL-8, and IFN-γ [37]. IL-6 is produced by neutrophils in response to *Cn* capsule size via interaction of GXM with complement [38]. It is possible that even when IL-6 enhances *Cn* capsule size, infected Wild-type mice were able to control the fungal burden in blood and brain because host phagocytes likely increased their Ab-mediated fungal phagocytic activity [39]. The clearance of *Cn* overlaps with the development of an adaptive immune response that enhances killing or containment of the fungi in granulomas [40]. Therefore, successful containment of the fungus requires both innate and adaptive immune responses. Interestingly, deletion of the Apt1 flippase in *Cn* reduces the packaging of GXM into extracellular vesicles and impairs GXM synthesis whereas it reduces the production of IL-6 during infection and CNS colonization [41]. The capsule size of IL-6 + anti-IL-6 cells were larger than those of untreated cryptococci, suggesting that the modulation of the capsule may be triggered by sensing or interacting with either the cytokine, Ab, or their complex.

We found that IL-6 deficiency resulted in different microglial morphology phenotypes in response to *Cn* infection particularly characterized by an abundance of dystrophic cells. The accumulation of dystrophic microglia is associated with tissue degeneration [42] or senescence [43]. Aging individuals with Down syndrome [43] and Alzheimer’s disease [44] present an increased number of dystrophic microglia, which are unable to carry out their homeostatic functions [43]. In fact, dystrophic microglia rather than activated microglia are present with tau pathology and may precede neurodegeneration in Alzheimer’s disease [44]. It is possible that the identification of dystrophic microglia in *Cn*-infected brain tissue may be a marker for the progression stage of cerebral cryptococcosis or cognitive decline in patients that recovered from the infection. In contrast, Wild-type and IL-6^−/−^ + rIL-6 brains had higher number of activated and phagocytic microglia in infected tissue, with these cell phenotypes surrounding the cryptococcomas or brain lesions, which are associated with stronger response to infection and tissue repair. It is important to highlight that of the different microglial phenotypes evaluated, IL-6^−/−^ + rIL-6 brains had ≥ 75% of activated and phagocytic microglia throughout the infection and may explain the longer survival of these mice even though they showed high fungal burden early. While Wild-type and IL-6^−/−^ brains exhibited a high percentage of ramified microglia early during the infection. For example, microglia cultured with rIL-6 or even neutralized rIL-6 demonstrated more phagocytosis and fungal killing than untreated cells (Fig. 9B). IL-6, in addition to IL-1β, TNF-α, and IL-12, is produced by microglia-like cells during interactions with acapsular or encapsulated strains of *Cn* [45]. Thus, it is plausible that IL-12 [46] and IL-6 [47] production by microglia stimulate a combination of T helper cells 1 and 2 (Th1 or 2), respectively, to facilitate engulfing of the yeast cells (Th2) and enhance killing of the fungus in the phagolysosome (Th1). Moreover, silencing IL-6 expression in microglia-like cells reduced phagocytosis of *Cn* cells after activation with LPS/IFN-γ (Fig. 9C). In addition to combat infection, microglia can be neuroprotective and may promote brain tissue repair in an IL-6-dependent manner [29] to alleviate the cognitive deficits arising from tissue injury in recovering patients from cryptococcal meningoencephalitis, although further studies are required to test this hypothesis.

We identified extensive microglia cell body debris in brain tissue from each experimental group, particularly in the boundaries of the regions with encephalomalacia. It is provocative to postulate that extensive *Cn* GXM secretion and its intimate interaction with microglia may cause the separation of these microglia cell body ramifications, which can accumulate in tissue and compromise the inflammatory responses. In this regard, HIV/AIDS patients with cryptococcal meningoencephalitis typically exhibit minimal inflammation due to their reduced number of CD4^+^ T cells [14]. Another possibility is that GXM induces the transition of microglia from ramified to dystrophic, compromising their effector functions. Future studies are necessary to validate these hypotheses and expand our insight on microglial responses to *Cn* CNS invasion and colonization.

Substantial astrocytosis and astrogliosis were observed in *Cn*-infected tissue slices from Wild-type and IL-6^−/−^ + rIL-6 mice. Considerable astrocyte reactivity was evident in Wild-type brain tissue and these cells produce high levels of IL-6, especially in traumatic brain injury [48]. Reactive astrocytes are found nearby large brain lesions or overlapping with fungal extracellular vesicles in human post-mortem tissue from individuals with HIV/AIDS [14] and mice [49], respectively, likely playing a critical and understudied role in containing infection [50]. Human astrocytes inhibit cryptococcal proliferation through activation of NO [51] and recently we showed that astrocyte NO activation is higher in astroglia than microglia [28]. Calcium binding-S100B protein is secreted by astrocytes and stimulates NO secretion by these glial cells in an autocrine manner [17]. Nevertheless, *Cn* can neutralize astrocyte derived NO without interfering with inducible NO synthase generation or catalytic activity [52]. We observed minimal astrogliosis in IL-6^−/−^ brain tissue infected with *Cn*; however, this was not surprising because these mice have previously shown impaired astroglia activation [53], indicating the importance of IL-6 in stimulating glia responses to fight this CNS mycosis.

Although studies by Li [22] and Blasi [24] had previously demonstrated the critical role of IL-6 in combating cerebral cryptococcosis, our study is unique relative to those seminal publications in that we used a validated clinical and highly virulent *Cn* (strain H99; serotype A; var. *grubii*) isolate. *Cn* invades the CNS in higher rates than *C. deneoformans* (serotype D; var. *neoformans*) and *C. gattii* (serotype B/C). Li and colleagues [22] used a *C. deneoformans* strain from unknown origin and low virulence, which explains why using a high inoculum (3.6 × 10^6^ CFU) to infect mice intranasally (i.n.) resulted in 90% survival rate of Wild-type C57BL/6 male mice and 100% mortality in IL-6^−/−^ mice after 120-dpi. In fact, C57BL/6 mice infected i.n. with 10^5^*Cn* H99 cells (a considerably lower inoculum) caused 100% mortality at 32-dpi [54], highlighting the importance of understanding the pros and cons of each study when making data analysis and interpretation. Another important discrepancy is that even though the IL-6^−/−^ mice in the Li’s study showed increased brain fungal burden as the disease progressed, the Wild-type mice had minimal cryptococcal brain invasion throughout the duration of the infection [22]. This is interesting because Coelho and colleagues have shown that H99 cryptococci rapidly (< 3 h) invades the brain after nasal infection [55]. Similarly, Blasi and colleagues [24] used the *Cn* (Sanfelice) Vuillemin (ATCC 11240) or *Cn* var. *shanghaiensis* Liao (described in 1980s) strain, an environmental isolate that is now classified as *C. gattii* [56]. *C. gattii* became prominent after a cryptococcosis outbreak on Vancouver Island, British Columbia, Canada particularly affecting individuals with apparent immunocompetency [57]. Although *C. gattii* causes pulmonary and CNS infection, *Cn* is still isolated from and associated with most of the cases of cryptococcosis (42.4% vs. 1.3%) in endemic regions such as sub-Saharan Africa [58]. Blasi also showed that exogenous and local (intracerebrally; i.c.) IL-6 supplementation of Wild-type mice prior to i.c. cryptococcal infection significantly reduced blood and brain fungal load, resulting in prolonged survival. Despite our study showing a similar result on the impact of IL-6 delaying mouse mortality, our findings are novel in that we not only corroborated the work by Li et al.. and Blasi et al., but further their observations by showing the importance of this cytokine on IL-6^−/−^ mice after exogenous systemic supplementation and on *Cn* by molecularly modulating its capsular production. Furthermore, this is the first time a specific study has been done on the effects IL-6 has on microglia and astrocyte responses to cerebral cryptococcosis in vivo, which is an important step forward to understanding the way *Cn* establishes and develops an infection in the brain environment.

**Fig. 10:**
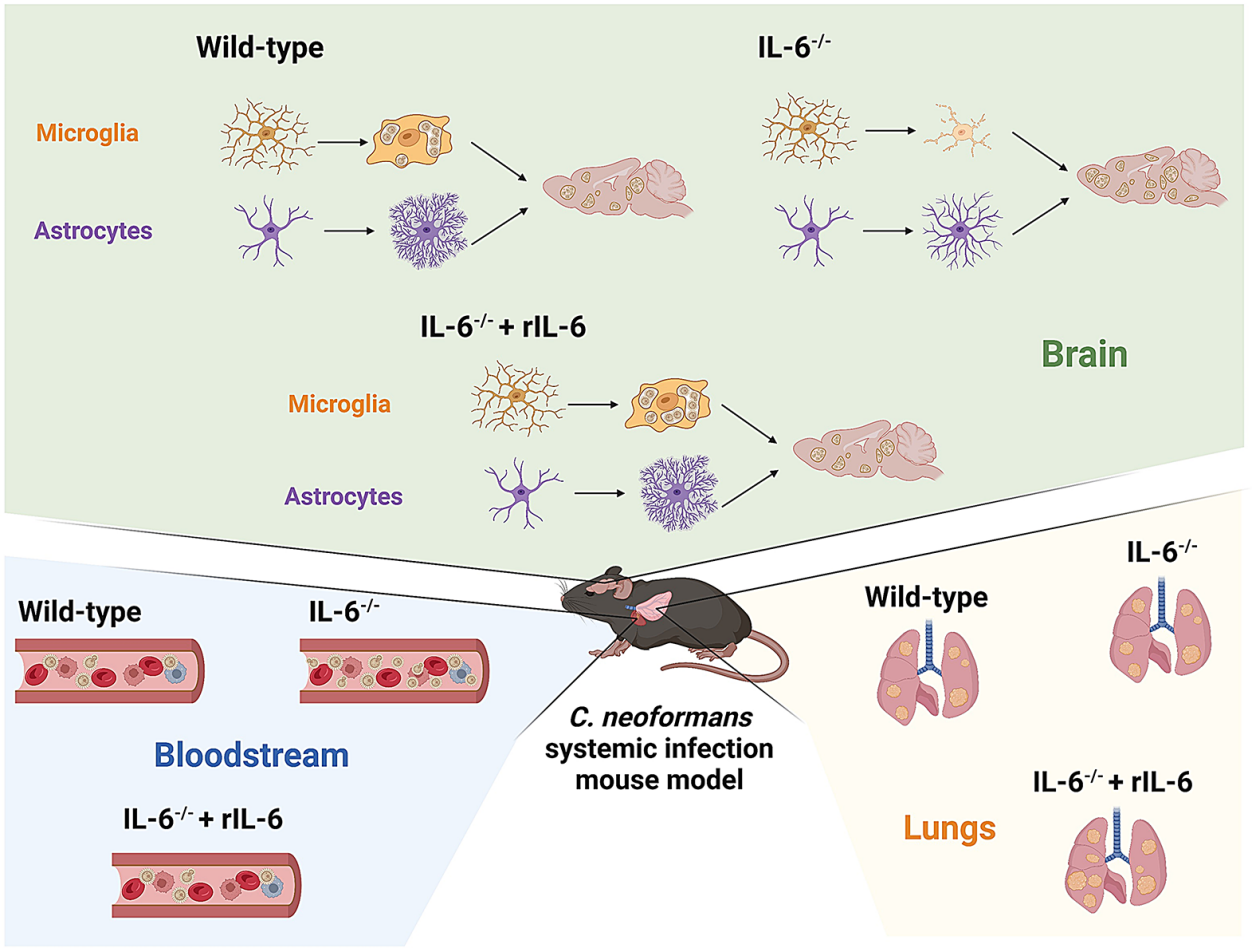
Model of role of IL-6 on *Cn* systemic infection. In vivo, the absence of IL-6 causes early inflammation but had no considerable effect in the mouse lungs as the infection progresses. However, *Cn* extensively proliferates in circulation of IL-6^−/−^ mice, thus being able to reach and colonize the CNS. In the brain, IL-6 stimulates astrogliosis and microglial cryptococcal phagocytosis and killing. The model was created with BioRender.com by Dr. Marta Reguera-Gomez

In conclusion, we have demonstrated that IL-6 has a significant role in combating *Cn* systemic infection in mice. The molecular mechanisms underlying this effect remain unknown. Our data suggest that IL-6 deficiency causes early inflammation but does not alter pulmonary disease as the infection progresses. IL-6 deficiency enhances cryptococcal proliferation in the bloodstream, which could potentially facilitate fungal crossing into the CNS, leading to alterations in glial activation and responses that are associated with increased mortality (Fig. 10). Further studies investigating the impact of immunomodulator molecules such as IL-6 on cerebral cryptococcosis are warranted for the development of antifungal therapy or treatments aimed to prevent, reduce, or manage *Cn* infections especially in at-risk immunosuppressed populations.

## Materials and methods

### *Cn*

*Cn* strain H99 (serotype A) was isolated and kindly provided by John Perfect at Duke University. Yeasts were grown in Sabouraud dextrose broth (pH 5.6; BD Difco) for 24 h at 30 °C in an orbital shaker (New Brunswick Scientific) set at 150 rpm (to early stationary phase). They were washed, counted, and resuspended in sterile 0.9% sodium chloride (saline) solution, USP (Baxter).

### NR-9460 cells

The murine microglial cell line NR-9460 (BEI Resources, NIAID, NIH) was derived from wild-type mouse brain tissue and immortalized by infection with the ecotropic transforming replication-deficient retrovirus J2. Characterization based on immunofluorescence, stimulation assays, and flow cytometry demonstrated that the NR-9460 cell line retains its glia-specific morphological, functional, and surface expression properties.

### Systemic infection and rIL-6 administration

Female C57BL/6 and B6.129S2-*Il6*^*tm1Kopf*^/J mice (IL-6^−/−^; 6 to 8 weeks old; 20 to 25 g; Jackson Laboratory) were injected in their tail vein with a 100-µL suspension containing 10^5^*Cn* H99 cells. The intravenous (IV) injection was selected due to its high reproducibility in *Cn* CNS infection models [28]. To mimic baseline physiological IL-6 levels [59], one-day prior to infection, a group of IL-6^−/−^ mice were injected IP with rIL-6 (1.6 pg/g) diluted in sterile saline solution. One-dpi, mice were injected with a 100-µL solution of sterile saline (untreated and IL-6^−/−^) or supplemented with rIL-6 (40 pg/g/day; [32]) IP every other day. The daily dose of rIL-6 was selected and calculated accordingly based on a previous report on cryptococcal infection that showed IL-6 serum concentrations of 500 pg/mL/mouse [60]. Animals were monitored for survivability or pathology (Fig. 1). The survival end points included inactivity, tachypnea, or loss of ≥ 25% of body weight from baseline weight. We monitored the mice twice daily for clinical signs (e.g., temperature), dehydration, and weight loss. Animals showing signs of dehydration or that lost more than 10% body weight received supportive care such as 1 mL of parenteral fluid supplementation (saline) and moist chow on the cage floor was provided.

### CFU determinations

Mice were bled from their facial vein using heparinized tubes for collection and euthanized at 3- and 7-dpi. Rapidly, lungs and brain were excised and weighed. The right-lung lobe and left-brain hemisphere tissues were homogenized in 5 mL of sterile phosphate-buffered saline (PBS, pH 7.3 ± 0.1). Blood and tissue homogenates were serially diluted, and a 100-µL suspension was plated on Sabouraud dextrose agar (BD Difco) and incubated at 30 °C for 48 h. Quantification of viable yeast cells from infected animals (*n* = 3–5/group/day) was determined by CFU counting of two dilutions per animal (*n* = 6 plates per fluid or tissue).

### Histopathology

The left-lung lobe and right-brain hemisphere were harvested and immersed in 4% paraformaldehyde (Fisher) overnight instead of perfusing the organs. Even though inflation of the lungs via intratracheal instillation of fixative is recommended to best preserve lung morphology and reduce artifactual atelectasis, this approach is contraindicated for lung infection as this can alter the anatomic location of fungal cells and cellular debris [61]. Then, tissues were washed 3X with sterile saline for 1 h, embedded in paraffin, 4 μm coronal sections were serially cut using a cryostat (Tanner Scientific, model: TN50), fixed onto glass slides, and subjected to hematoxylin-eosin (lung) or mucicarmine (brain) staining (*n* = 3 mice per group) to examine host tissue or fungal morphology, respectively. GXM (MAb 18B7 is an anti-cryptococcal GXM IgG1 generated and generously provided by Arturo Casadevall at the Johns Hopkins Bloomberg School of Public Health; 1:1,000 dilution), Iba-1 (rabbit anti-human Iba-1; 1:1,000 dilution; FujiFilm Wako), and GFAP (rabbit anti-human GFAP 2033X; 1:2,000 dilution; Dako) specific Ab (conjugated to horseradish peroxidase; dilution: 1:1,000; Santa Cruz Biotechnology) immunostaining to assess capsular release, microglial phenotype, and astrocyte morphology, respectively, near cryptococcomas. The slides were visualized blindly by three independent investigators using a Leica DMi8 inverted microscope, and images were captured with a Leica DFC7000 digital camera using LAS X digital imaging software. The morphology of microglia and astrocytes in brain tissue was quantified (*n* = 3 mice per group) by LAS X digital imaging software using the recorded 40X images and standardized 250 × 250-µm^2^ squares near cryptococcomas. GXM distribution in tissue sections at 10X magnification (*n* = 10–15 fields per brain region; *n* = 3 mice per group) was calculated using NIH ImageJ color deconvolution tool software (version 1.53q). The mean color intensity of the GXM for each treatment group was plotted in Prism 10.1.2. (GraphPad). The images were also examined, analyzed, and described by Dr. Mohamed F. Hamed, a veterinary pathologist.

### Brain IL-6 determinations

Brain tissue (0.2 g) was placed in 1.8 mL of RIPA buffer and homogenized, and the supernatant was stored at − 20 °C until analyzed. An enzyme-linked immunosorbent assay (ELISA) was performed using the Preprotech kit for each cytokine following the manufacturer’s protocol. Briefly, microtiter polystyrene plates were coated with capture anti-IL-6 (1 µg/mL) overnight and incubated at 4 °C. Next day, each well was blocked with 1% bovine serum albumin in PBS for 2 h at room temperature (RT). Next, the brain samples were serially diluted on the plate using a multi-pipette and incubated overnight at 4 °C. The ELISA was completed by adding 0.5 µg/mL of a specific detection Ab followed by a 2 h incubation at RT, then by avidin-horseradish peroxidase conjugate (HRP; 1:2,000 dilution) for 30 min at RT, and finally revealing by adding 2,2′-azinobis (3-ethylbenzothiazoline-6-sulfonic acid)-diammonium salt substrate. In each step, the wells were washed with 0.05% Tween 20 in PBS. The optical density was assessed at 450 nm using a BioTek Synergy LX Multimode Reader and monitored every 10 min for 1 h. Each sample was tested in triplicates in two independent ELISA measurements.

### Confocal microscopy

For immunofluorescence studies, yeasts were incubated in 96-well microtiter plates with glass bottom for 1 h at 30 °C with FUN-1 (green/red; 10 µM; 470/590 nm; Invitrogen), washed 3 times with PBS, and incubated with MAb 18B7 (2 µg/mL) for 1 h at 37 ºC, followed by allophycocyanin-conjugated goat anti-mouse IgG1 (blue; 651/660 nm; Thermo Fisher). Pictures were taken in a Nikon Eclipse Ti2 Inverted confocal microscope at 20X magnification and processed with NIS-Elements Imaging software.

### Capsule measurements with India ink

The capsule volume of *Cn* cells cultured in the absence (untreated) or presence of rIL-6 or rIL-6 + anti-IL-6 for 24 h was measured. To observe and measure the size of the capsule, 10 µL of each cell suspension were mixed with an India Ink (BD) drop and the capsules visualized under light microscopy. Images were randomly taken with a Leica DMi8 inverted microscope and DFC7000 T digital camera. The diameters of both capsule and cell body were measured using the Leica software platform LAS X. Capsule volume was calculated using the volume formula, where R = radius of capsule and r = radius of cell body: capsule volume (V) = 4/3 π (R^3^ – r^3^). Fifty cells were analyzed per condition.

### RNA extraction and cDNA synthesis

RNA extraction was performed at 2 and 24 h after incubation of cryptococci in absence or presence of either rIL-6 or rIL-6 + anti-IL-6 using the Quick-RNA fungal/bacterial extraction kit (Zymo Research), following the manufacturer’s instructions. To remove any genomic DNA carryover, the samples were treated with DNase I (Zymo Research) for 15 min at RT, followed by RNA recovery in DNase/RNase free water. Finally, 200 ng of total RNA were used to synthesize cDNA using the Verso cDNA Synthesis kit (Thermo Fisher), following the manufacturer’s instructions. The control reaction was set up using all components of the reaction mixture but without RNA sample.

### qPCR

The genes selected for quantification were *Cap59* [26] and *Grasp* [27], both involved in capsule synthesis. The primers and annealing temperatures used for qPCR analysis are described in Table 1. The expression of genes was determined by qPCR using PowerUp Syber Green Master Mix 2x (Applied Biosystems). Reactions were set up using 250 nM primers and 4.5 µL of the cDNA template (diluted 1:10). The cycling conditions used were as follows: 50 °C for 2 min, 95 °C for 2 min, and then 40 amplification cycles of 95 °C for 20 s, 52–56 °C for 30 s, and 72 °C for 30 s. The samples were cooled to 55 °C, and a melting curve for temperatures between 55 and 95 °C, with 1 °C increments, was recorded. Relative expression was determined using the 2-^DDCT^ method on a qTower thermocycler (Analytik Jena). A non-template for qPCR was used as control and all reactions were carried out in triplicate. Target gene expression was measured using expression relative to that of the glyceraldehyde-3-phosphate dehydrogenase (GAPDH) reference gene as well as the untreated cryptococci as reference sample.

**Table 1:** Primers used in the qPCR studies

Gene	Annealing temperature (°C)	Primer Sequence (5’-3’)
*Cap59*	54	Forward: GAGTGTCTCCGCAACCCGCA Reverse: TACTTGTGCTGCCTCGCCTG
*Grasp*	54	Forward: AGTTCTTTACCCTACTAGACAG Reverse: TCTCCTCACATTGTCAGATTC
*GAPDH*	56	Forward: GCTGCYGCTAACATCATCCC Reverse: YGAAATCAGTRGAGACAACA

### *Cn* growth assessment

Fungal growth (10^6^ cells) in minimal medium without (untreated) or with rIL-6 (10 ng/mL) or rIL-6 (10 ng/mL) + anti-IL-6 (20 ng/mL) was assessed in real-time at an optical density (OD) of 600 nm every 30 min using a microplate reader (Bioscreen C; Growth Curves USA). Ten replicates were analyzed per condition.

### Cell transfection assay

siRNA transfection of NR-9640 cells was performed with a pre-designed mouse siRNA IL-6 (Invitrogen) and a siRNA negative control (Invitrogen). One day prior transfection, NR-9640 cells were equally placed in 12- or 24-well plates at a confluency of 70% and incubated in Opti-MEM I reduced serum supplemented with 5% FBS at 37°C and 5% CO_2_. On the day of the transfection, Opti-MEM I reduced serum medium without FBS was used to prepare the mix of the Lipofectamine™ RNAiMAX Transfection Reagent (Invitrogen) and each siRNA at a final concentration of 50 nM followed by an incubation at RT for 10 min. The mixture was then added to the microglia-like cells and incubated for 24 h at 37°C and 5% CO_2_. Reverse transfection was also performed by preparing the complexes inside the wells and then adding cells and medium, according to the manufacturer’s protocol. Transfection efficiency was detected after 24 h and an additional 2 h treatment with activation media [Opti-MEM I with 2% FBS and supplemented with LPS (0.5 µg/mL)/IFN-γ (5 ng/mL) to stimulate the production of IL-6. Relative IL-6 expression was measured via qPCR using the following specific oligonucleotides: forward 5’CACGGCCTTCCCTACTTCAC3’ and reverse 5’TGCAAGTGCATCATCGTTGT3’. RNA extraction and cDNA synthesis after siRNA treatment were performed using the Quick-RNA MiniPrep Plus (Zymo Research), following the manufacturer’s instructions. To remove any genomic DNA carryover, the samples were treated with DNase I (Zymo Research) for 15 min at RT, followed by RNA recovery in DNase/RNase free water. Finally, 100 ng of total RNA were used to synthesize cDNA using the Verso cDNA Synthesis kit (Thermo Fisher), following the manufacturer’s instructions. Two control reactions were set up using all components of the reaction mixture but without RNA sample or reverse transcriptase. The IL-6 protein levels were measured in the supernatant of NR-9460 cell culture 24 h after activation by ELISA as described. Activated NR-9460 cells with LPS/IFN-γ without any siRNA but exposed to the Lipofectamine RNAiMAX Transfection Reagent were used as a reference of IL-6 expression/secretion.

### Phagocytosis assay

Monolayers of NR-9640 cells were washed thrice with PBS, and Dulbecco’s Modified Eagle Medium (DMEM; feeding medium; [62, 63]) supplemented with IFN-γ (5 ng/mL) and LPS (0.5 µg/mL) was added, followed by the addition of fluorescein isothiocyanate (FITC)-stained (1 h at RT, shaking) pre-opsonized cryptococci with MAb 18B7 (10 µg/mL) for 1 h, in a microglia : cryptococci ratio of 1:10 (5 × 10^5^ : 5 × 10^6^ cells). The plates were incubated for 4 h at 37 °C and 5% CO_2_ for phagocytosis. For phagocytosis assessment, the monolayer coculture was washed thrice with PBS to remove nonadherent cells, trypsinized for 1 min to detach microglia, centrifugated, and resuspended in FACS buffer. Cells were strained through a 100 μm filter, stained with 7-aminoactinomycin D (7-AAD; Invitrogen) for viability, and fixed. Samples were processed on a BD Accuri C6 flow cytometer and *Cn* phagocytosis by microglia was analyzed using the FlowJo software. The percentage of phagocytosis per sample was determined by gating phagocytic microglia showing fluorescence at 516 nm after ≥ 10,000 events.

### Killing assay

After Ab-mediated phagocytosis, each well containing interacting microglia-cryptococci was gently washed with feeding medium three times to get rid of fungal cells which were not phagocytized. Then, cryptococcus-engulfed microglia were incubated for 24 h at 37 °C and 5% CO_2_. Microglia-like cells were lysed by forcibly pulling the culture through a 27-gauge needle 5 to 7 times. A 100-µL volume of suspension containing cryptococci was aspirated from the wells and transferred to a microcentrifuge tube with 900 µL PBS. For each well, serial dilutions were performed and plated in triplicate onto Sabouraud dextrose agar plates, which were incubated at 30 °C for 48 h. Viable cryptococcal cells were quantified as CFU. Although it is plausible that noninternalized cryptococci could replicate for several generations in feeding medium during a 24-h period, wells for each condition were microscopically monitored after phagocytosis to reduce the possibility of obtaining confounding results.

### Statistical analysis

All data were subjected to statistical analysis using Prism 10.1.3 (GraphPad). Differences in survival rates were analyzed by the log-rank test (Mantel-Cox). *P* values for multiple comparisons were calculated by one-way analysis of variance (ANOVA) and were adjusted by use of the Tukey’s *post hoc* analysis. *P* values of < 0.05 were considered significant.

## Electronic supplementary material

Below is the link to the electronic supplementary material.

Supplementary Material 1: Wild-type, IL-6^−/−^, IL-6^−/−^ + rIL-6 mice systemically infected with *Cn* showed no difference in pulmonary pathology development. Mice were infected IV with 10^5^ cryptococci and euthanized at (A) 3- and (B) 7-dpi. Representative images of lung tissue sections stained with periodic acid-Schiff. Black, yellow, blue, and green arrows indicate normal, atelectasis, hyperplasia, and cryptococcoma formation, respectively. Red arrowheads denote bronchus-associated lymphoid tissue. Top, middle, and bottom panels indicate 4, 10, and 20X magnification, respectively. Scale bars: 50 μm.

Supplementary Material 2: Wild-type mice infected with *Cn* show increased IL-6 levels in the brain compared to uninfected mice. The supernatants from uninfected and H99-infected Wild-type (C57BL/6) brains at 3- and 7-dpi were processed and analyzed for IL-6 levels by ELISA. Bars represent the mean values and error bars indicate SDs. Each circle represents supernatant from an individual brain (*n* = 3 supernatants per group). Significance (****, *P* < 0.0001; ***, *P* < 0.001; **, *P* < 0.01) was calculated by one-way ANOVA and adjusted using Tukey’s post-hoc analysis. ns denotes comparisons which are not statistically significant. Cytokine quantification was performed twice with similar results obtained.

Supplementary Material 3: siRNA treatment does not affect NR-9460 microglia-like cell viability. (A) Representative flow cytometry dot plots for untreated and siRNA IL-6- or siRNA negative control-treated NR-9460 cells are shown. Cells were stained with 7-AAD for viability after a 24 h siRNA treatment at 37°C and 5% CO_2_. Each plot was generated after ≥ 10,000 events were analyzed. (B) The percentage of microglia-like cell viability was determined. Each symbol represents an independent replicate (*n* = 4). Bars and error bars denote means and SDs, respectively. Significance (*, *P* < 0.05) was calculated by one-way ANOVA and adjusted using Tukey’s post-hoc analysis. ns denotes comparisons which are not statistically significant.

## Data Availability

No datasets were generated or analysed during the current study.
