# Supplementary figures and images for: IL-6 deficiency accelerates cerebral cryptococcosis and alters glial cell responses
Source: J Neuroinflammation. 2024 Sep 27;21:242. doi: 10.1186/s12974-024-03237-x (PMC11437997; doi:10.1186/s12974-024-03237-x)

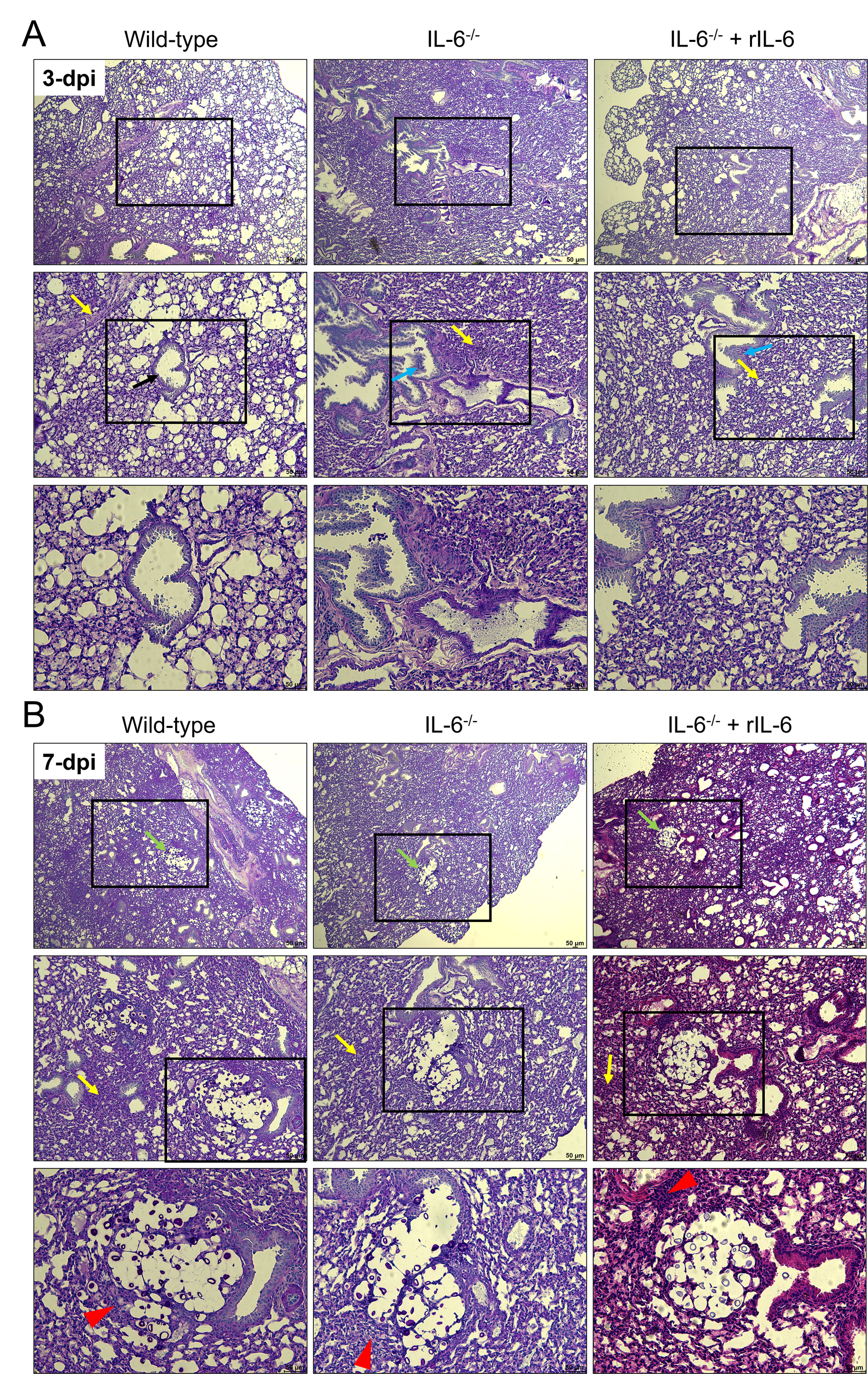

Supplement: Supplementary file 1 — Supplementary Material 1: Wild-type, IL-6−/−, IL-6−/− + rIL-6 mice systemically infected with Cn showed no difference in pulmonary pathology development. Mice were infected IV with 105 cryptococci and euthanized at (A) 3- and (B) 7-dpi. Representative images of lung tissue sections stained with periodic acid-Schiff. Black, yellow, blue, and green arrows indicate normal, atelectasis, hyperplasia, and cryptococcoma formation, respectively. Red arrowheads denote bronchus-associated lymphoid tissue. Top, middle, and bottom panels indicate 4, 10, and 20X magnification, respectively. Scale bars: 50 μm. [file 12974_2024_3237_MOESM1_ESM.tif]

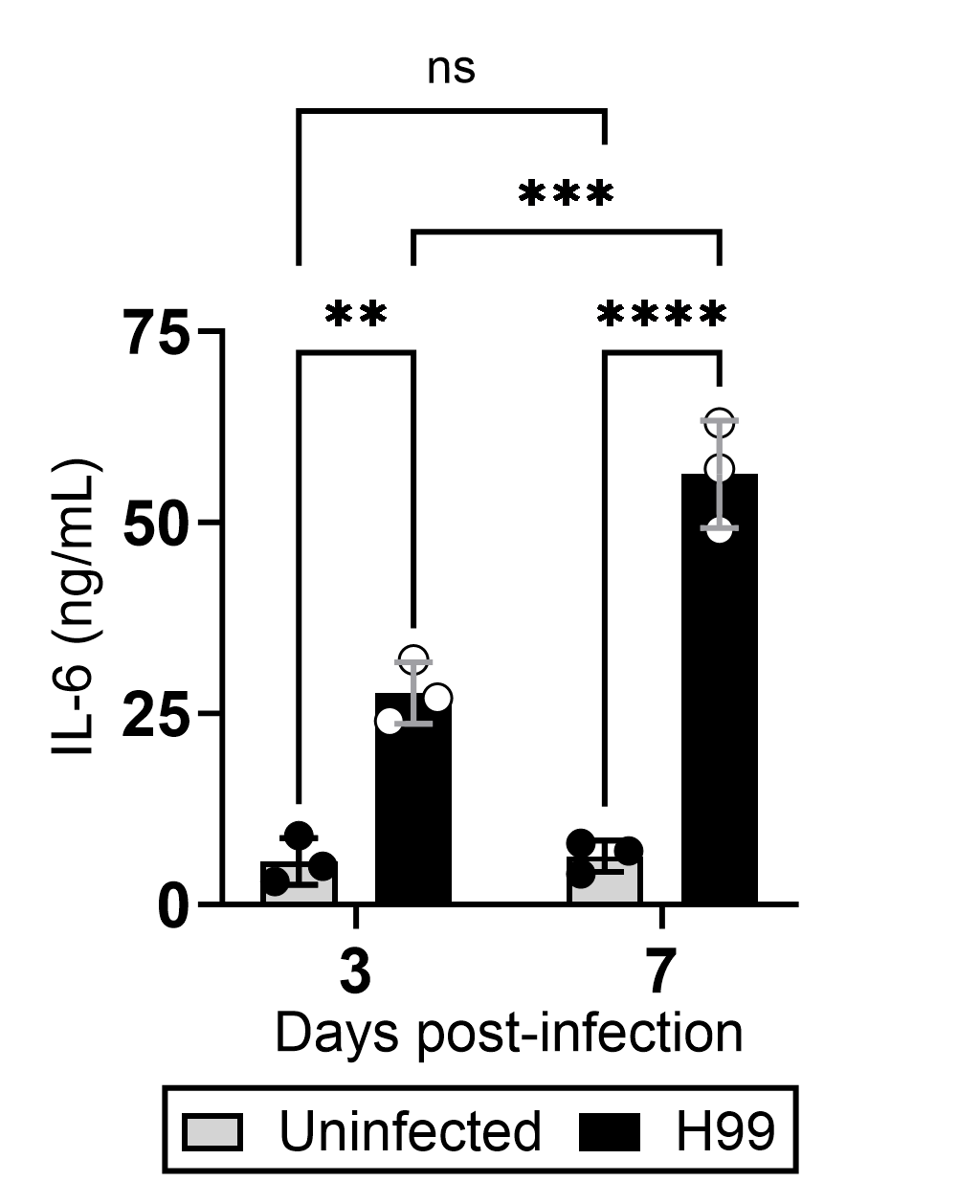

Supplement: Supplementary file 2 — Supplementary Material 2: Wild-type mice infected with Cn show increased IL-6 levels in the brain compared to uninfected mice. The supernatants from uninfected and H99-infected Wild-type (C57BL/6) brains at 3- and 7-dpi were processed and analyzed for IL-6 levels by ELISA. Bars represent the mean values and error bars indicate SDs. Each circle represents supernatant from an individual brain (n = 3 supernatants per group). Significance (****, P < 0.0001; ***, P < 0.001; **, P < 0.01) was calculated by one-way ANOVA and adjusted using Tukey’s post-hoc analysis. ns denotes comparisons which are not statistically significant. Cytokine quantification was performed twice with similar results obtained. [file 12974_2024_3237_MOESM2_ESM.tif]

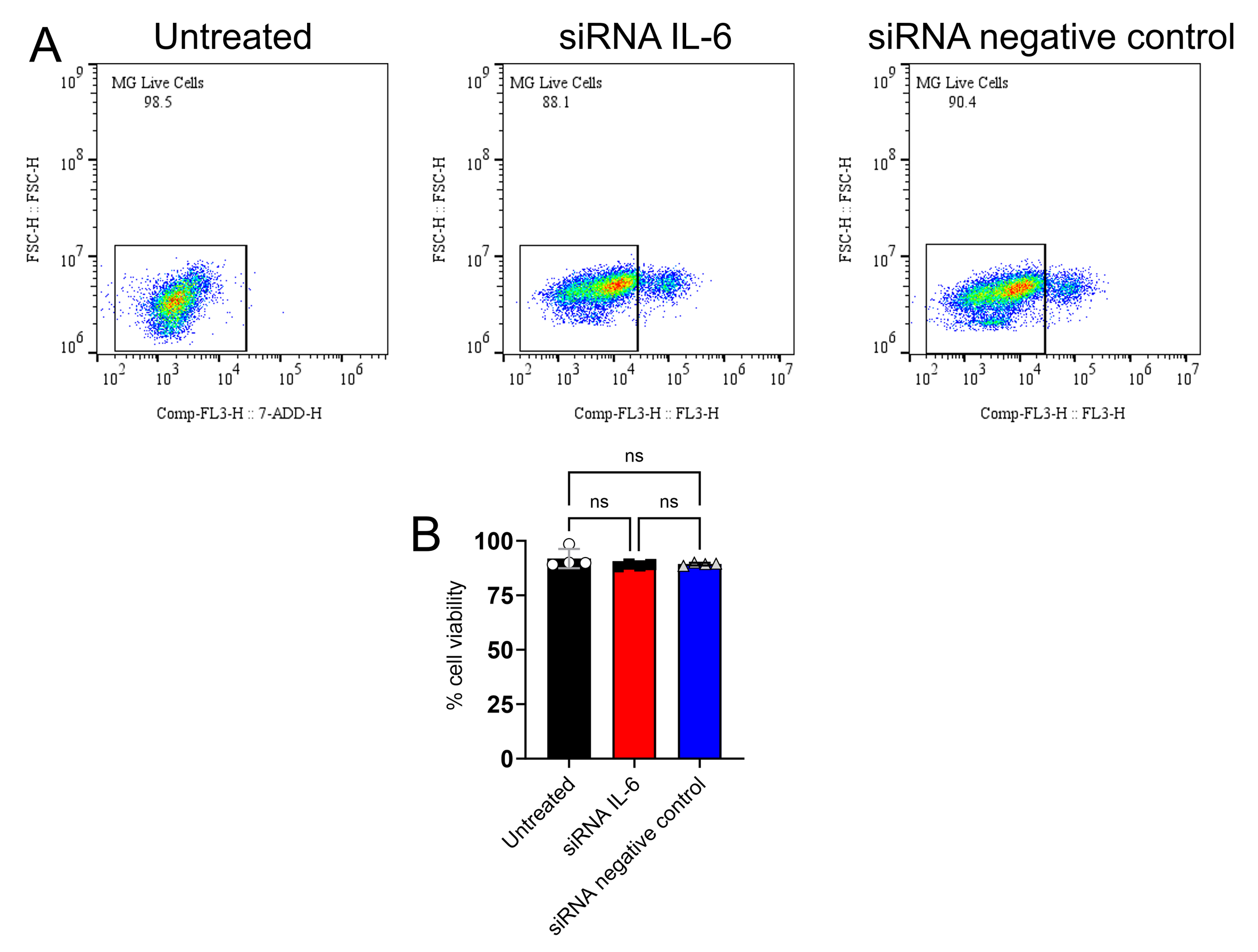

Supplement: Supplementary file 3 — Supplementary Material 3: siRNA treatment does not affect NR-9460 microglia-like cell viability. (A) Representative flow cytometry dot plots for untreated and siRNA IL-6- or siRNA negative control-treated NR-9460 cells are shown. Cells were stained with 7-AAD for viability after a 24 h siRNA treatment at 37°C and 5% CO2. Each plot was generated after ≥ 10,000 events were analyzed. (B) The percentage of microglia-like cell viability was determined. Each symbol represents an independent replicate (n = 4). Bars and error bars denote means and SDs, respectively. Significance (*, P < 0.05) was calculated by one-way ANOVA and adjusted using Tukey’s post-hoc analysis. ns denotes comparisons which are not statistically significant. [file 12974_2024_3237_MOESM3_ESM.tif]
